# Using Vessel Monitoring System Data to Identify and Characterize Trips Made by Fishing Vessels in the United States North Pacific

**DOI:** 10.1371/journal.pone.0165173

**Published:** 2016-10-27

**Authors:** Jordan T. Watson, Alan C. Haynie

**Affiliations:** 1 NOAA / National Marine Fisheries Service, Alaska Fisheries Science Center, Auke Bay Laboratories, Juneau, Alaska, United States of America; 2 School of Fisheries and Ocean Sciences, University of Alaska Fairbanks, Juneau, AK, United States of America; 3 NOAA / National Marine Fisheries Service, Alaska Fisheries Science Center, Resource Ecology and Fisheries Management Division, Seattle, Washington, United States of America; Aristotle University of Thessaloniki, GREECE

## Abstract

Time spent fishing is the effort metric often studied in fisheries but it may under-represent the effort actually expended by fishers. Entire fishing trips, from the time vessels leave port until they return, may prove more useful for examining trends in fleet dynamics, fisher behavior, and fishing costs. However, such trip information is often difficult to resolve. We identified ~30,000 trips made by vessels that targeted walleye pollock (*Gadus chalcogrammus*) in the Eastern Bering Sea from 2008–2014 by using vessel monitoring system (VMS) and landings data. We compared estimated trip durations to observer data, which were available for approximately half of trips. Total days at sea were estimated with < 1.5% error and 96.4% of trip durations were either estimated with < 5% error or they were within expected measurement error. With 99% accuracy, we classified trips as fishing for pollock, for another target species, or not fishing. This accuracy lends strong support to the use of our method with unobserved trips across North Pacific fisheries. With individual trips resolved, we examined potential errors in datasets which are often viewed as “the truth.” Despite having > 5 million VMS records (timestamps and vessel locations), this study was as much about understanding and managing data errors as it was about characterizing trips. Missing VMS records were pervasive and they strongly influenced our approach. To understand implications of missing data on inference, we simulated removal of VMS records from trips. Removal of records straightened (i.e., shortened) vessel trajectories, and travel distances were underestimated, on average, by 1.5–13.4% per trip. Despite this bias, VMS proved robust for trip characterization and for improved quality control of human-recorded data. Our scrutiny of human-reported and VMS data advanced our understanding of the potential utility and challenges facing VMS users globally.

## Introduction

Fisheries researchers often use catch per unit effort (CPUE) as a means by which to assess the dynamics and health of fish stocks. In such cases, effort is typically defined as the time during which fishing gear is actively deployed, and thus CPUE becomes a standard metric for resolving the costs of fishing on commercial stocks. Resolving the costs of fishing to humans, however, relies not only upon how long gear was deployed, but also upon how long a vessel remained at sea; and how far and where it traveled [1]. Such fundamental aspects of fishing trips (e.g., duration, distance traveled, location) become increasingly critical as we consider the impacts on fishers’ from a changing climate (e.g., [2]), shifting fish populations (e.g., [3]) and variable fuel costs (e.g., [4]). These factors may affect the profitability of trips, so as fishers strive to minimize cost, the ability to assess changes in trip characteristics may be fundamental for understanding fleet dynamics over time. Despite the importance of resolving trip behaviors, the details of fishing trips often remain poorly characterized, or insufficient data may be available to examine their trends.

A “fishing trip” is one of the simpler concepts in fisheries research but in practice, both the data and even the definition can be rather complex. There are many definitions of a fishing trip that may affect the interpretation of vessel behavior. In the United States, regulations define trips based on management programs and vessel classifications, so a statutory “trip” can have different meanings (50 CFR 679.2). For example, regulations specify that a trip begins for catcher vessels targeting groundfish when the harvesting of fish commences; the trip ends when the last of the catch is offloaded. This definition may drastically underestimate the time that a vessel spends at-sea and it may provide no guidance for determining, for example, whether fishers now travel farther to catch their fish than in previous years. The North Pacific Groundfish Observer Program (NPGOP) starts a trip when a vessel unties from a dock or floating processor and ends a trip when the vessel ties up at either a dock or a floating processor, or if an observer exits the vessel [5]. Observers in the NPGOP have been present on many of the trips in the Bering Sea and Aleutian Islands (BSAI) and the Gulf of Alaska (GOA) for more than a quarter century. However, detailed trip information has not always been maintained and the levels of coverage have varied across fleets and years. Thus, while the trip definition used by the NPGOP is largely conducive to examining trip behaviors over time, the sampling extent may leave trip patterns incomplete for both fishing and non-fishing trips in the region. In such cases, vessel monitoring systems (VMS) have the potential to resolve substantial uncertainty in vessel trips, from the time a vessel leaves a port / processor to the time it returns to a port/ processor. Thus, it is this definition of a fishing trip (from port / processor to port / processor) that we use throughout our study.

VMS are increasingly required for fishing fleets worldwide. Largely implemented to enforce fishery closures or other spatial management regulations, VMS transmit a vessel’s location (latitude and longitude) at regionally-mandated time intervals, typically from 30–120 min. Supplemental to their utility for law enforcement, VMS data have been used to estimate fishing effort (e.g., [6–7]), validate logbook data (e.g., [8–9]), and delineate habitats impacted by fishing (e.g., [10–12]). Such applications of VMS data can be applied to cases when vessels are either observed or unobserved, and they can also be used to resolve gaps in data resulting from sparse observer coverage. Several software packages (VMStools [13]; VMSbase [14]) even provide automated analyses of some of the above functions with VMS data, but they are refined primarily for European fleets and ports, leaving limited functionality for U.S. and other non-European fisheries. This is not surprising, however, as VMS data from U.S. fishing vessels, for example, have only been sparsely used in research [9,15] despite the U.S. having more vessels with VMS (> 4,000) than any other nation (www.nmfs.noaa.gov/ole/about/our_programs/vessel_monitoring.html). With tens of millions of VMS records for some U.S. fisheries, these data represent a major source of information for fisheries management that has been largely under-utilized. As such, the limitations of these data have also been scarcely addressed in the United States. In theory, VMS records should make trips easy to identify. Trips begin when a vessel leaves a port/ processor and they end when the vessel returns to a port/ processor. However, inconsistencies in the transmission of VMS data, variable port geography and fishing behaviors, vessels delivering to multiple ports / processors, and other possible factors complicate trip identification.

We present an example using a VMS dataset that has not previously appeared in the literature. Thus, we provide a framework for VMS data whose utility and limitations were previously unknown, a situation that is applicable to many VMS programs worldwide. The fishery for walleye pollock (*Gadus chalcogrammus*; hereafter “pollock”) in the eastern Bering Sea is the largest commercial fishery in the United States. The fishery was rationalized (i.e., moved to catch shares) by the American Fisheries Act (AFA) in 1998 (www.npfmc.org/american-fisheries-act-afa-pollock-cooperatives/), and it has an annual harvest valued at more than $1 billion [16]. The pollock quota is divided roughly in half between at-sea catcher-processors and catcher-vessels (CVs) that deliver to both shoreside processors and “mothership” vessels. Our study examines CVs in particular, whose pollock trips are usually 1–4 days long but whose non-pollock trips span the North Pacific and may last up to several weeks. While these vessels are the only catcher boats permitted to fish for pollock in the Bering Sea, many of these vessels also participate in other fisheries (including non-trawl fisheries) from the Bering Sea to the west coast of the United States (a range of ~ 4,000 km). It is because of their participation in the pollock fishery that they have been required to transmit VMS data since 2002. However, because of their broad spatial extent and participation in many fisheries, these vessels also offer a good proxy for understanding vessel movements into and out of more than 50 fishing ports in the North Pacific, as many of them spend extended periods on non-pollock trips as well as pollock trips.

Our objectives were to develop a VMS-based modeling approach to (1) identify individual BSAI and GOA trips made by CVs from the Bering Sea pollock fleet; (2) quantify trip distances and durations traveled and ground-truth them against observer data; (3) characterize trips as “fishing for pollock,” “fishing for other target species,” or “non-fishing”; (4) identify ways that autonomously-collected data like VMS data may be used to corroborate, and to quality-check human-collected sources like observer and fish ticket (fishery landings reports) data. We present our method, refined for the North Pacific, as a demonstration of how these data may be approached, but the generalities of our methodology and the data issues identified are applicable to many global fisheries with VMS.

## Methods

We first present an overview of the data, followed by a description of the algorithm used to identify individual trips (including calculation of fields, algorithmic rules, and integration of data sources). We then describe the calculation of trip metrics (distance and duration) and we use trip duration to compare VMS-based trips with observed trips. Next, we use a set of decision rules and regressions to characterize trips as fishing versus non-fishing and to identify the type of fishing when it occurs. Detailed appendices for methodological specifics are provided to assist researchers using VMS that face similar data challenges. However, the core of this section is written more generally to accommodate users of different VMS datasets. All analyses were performed using R Statistical Software Version 3.2.1 [17], with specific packages as noted in the text.

### Data overview and preliminary processing

All available data from each of three sources were extracted from their respective databases (VMS [18]; Observer [19]; Fish ticket [20]) for any CV that was permitted to fish in the AFA pollock fishery from 2008–2014 (Table 1). Each of these datasets are confidential and their access requires written authorization from their respective entities within National Oceanic and Atmospheric Administration’s National Marine Fisheries Service (NOAA Fisheries) and the State of Alaska.

**Table 1:** Description of data coverage and sources (see references). Data coverage has varied over time. Since 2011, pollock vessels have been fully observed; previously, vessels < 125 feet long were only observed for 30% of pollock fishing days at sea while longer vessels were fully observed.

Data	Coverage requirement	Years	Vessels	N	Source
VMS	100% of trips	2008–2014	91	~3.5 million VMS records*	18
Observer	30% of pollock fishing days at sea	2008–2010	65	2,366 trips^†^	19
Observer	100% of pollock fishing days at sea	2008–2010	26	1,897 trips^†^	19
Observer	100% of pollock fishing days at sea	2011–2014	91	14,482 trips^†^	19
Fish ticket	100% of fishing trips	2008–2014	91	27,503 trips	20

* Individual VMS records

^†^ Trips with observed durations > 200 min.

VMS have been mandated to transmit the location of vessels fishing for pollock in the BSAI at 30 min intervals since 2002. However, as this paper relies on a comparison with observer data to validate our approach, we present only those years with the requisite trip information (e.g., start and stop times) from observers (2008–2014). VMS data are required to be transmitted continuously, including when vessels are in port, though exceptions occasionally occur during extended port or anchorage periods. Preliminary processing of VMS data were required before project objectives could be addressed. Duplicate VMS records were removed and several data fields were generated: distance between sequential VMS records for a given vessel, distance from port, vessel speed, and the State and federal management areas for each VMS record (see S1 Text for descriptions of field calculations). VMS records were occasionally reported for which a vessel position was egregiously distant from its nearest neighbors, resulting in nonsensical vessel speeds or locations (e.g., on-land). Maximum vessel speeds were typically ~ 12 knots (22.2 kph) so we removed all VMS records with apparent speeds > 14 knots (a conservative upper bound) to remove erroneous records. VMS data were linked to available observer data such that a VMS record was part of an observed trip if its time-stamp fell within the observer-recorded start and stop time for a trip.

Throughout the study period, CVs with VMS have been monitored by government-trained observers through the NPGOP (part of NOAA Fisheries, Alaska Fisheries Science Center). Observer coverage of CVs was divided into two components: vessels with 100% coverage of pollock fishing days at sea (≥125 ft. in length) and vessels with historically only 30% coverage (< 125 ft. in length). Prior to 2007, no trip data (haul-level data only) were collected for the fleet and, for our purposes, a trip was unobserved if it lacks trip information (e.g., start and stop times). From late 2007 through 2010, trip records were maintained for all observed trips. Beginning in 2011, 100% of federally managed fishing days at sea for the entire pollock fleet became observed, creating a complete record of trips for all pollock fishing activity in the Bering Sea since that time. However, even for 100% observed vessels, some trips remained unobserved or may lack detailed trip information because they were not part of a federally regulated fishery that required observer coverage. For example, vessels may be chartered for research, participate in state-waters fisheries, transport salmon catch between smaller vessels and processors (“tendering”), or undergo long transits to ports outside of Alaska. Furthermore, as observer data do not indicate a vessel’s target species, those data alone may be insufficient to indicate whether a vessel was fishing for pollock (some CVs also target crab or other groundfish).

A potential data source for identifying target species and characterizing the type of fishing trip is fish ticket data. Seafood processors issue fish tickets when CVs land their catches and they record the date during which catches by species are landed. Additionally, fishers report the gear type (e.g., pelagic or bottom trawl, longline, pot), a code that identifies the management / permit program under which fishing occurs, and the management areas in which vessels report fishing. The data available from these landings data have evolved over time and some fields may not be present in all years.

### Trip identification algorithm

To address our first objective, we developed an algorithm that partitioned strings of consecutive VMS records for each vessel into individual trips. The foundation of the trip algorithm was to identify when vessels transitioned from being *at-sea* to being *in-port* and when they transitioned to being *at-sea* once again. For the majority of trips (88%), this simple approach (similar to that of [13–14]) was sufficient. However, due to missing VMS records, the identification of vessels’ transitions into and out of ports sometimes required a number of nuanced steps.

Initial assignment of *in-port* designations was made for all VMS records within 10 nautical miles (nmi) (18.5 km) of the nearest port. Dutch Harbor and Akutan—the primary ports for the AFA pollock fishery—sit within large protected bays where relatively little fishing occurs; an examination of VMS data confirmed that few vessels traveled within 10 nmi of either port unless the port was their destination (or origin). In contrast to Dutch Harbor and Akutan, many of the smaller, more exposed ports were located < 10 nmi from fishing grounds or vessel transit corridors so their *in-port* definitions were individually constrained to port-specific distances < 10 nmi. Due to gaps in VMS coverage, simple port polygons (like those used by [13]) were not always sufficient for identifying when a vessel was returning to port. Combinations of distance from port, vessel speed and the amount of time between VMS records were often necessary to resolve whether vessels were leaving/ entering port, fishing or simply passing a port (S2 Text).

#### Fish ticket matching

Fish processors issue fish tickets to vessels when they deliver their catch. Fish tickets, observer, and VMS data all share a vessel identification number which bolsters the ability to join the datasets. The utility of matching VMS-based trips to fish tickets was threefold: identification of missing port information, distinguishing between fishing and non-fishing trips, and determining if fishing trips were AFA pollock trips.

Long gaps between VMS records could obscure a trip’s *in-port* periods, and thus the port of embarkation or disembarkation may be unknown. However, when a fish ticket could be matched to the VMS trip, it identified the port in which the trip ended. In some cases, the fish ticket match could also elucidate missing ports of embarkation, though these matches were less obvious as embarkation port was not recorded on the fish ticket.

Fish tickets included fields for the date that fishing started within each management area and the date on which those fish were landed. The procedure to match fish tickets to VMS relied on whether a trip included at least one VMS record that fell within a reported state statistical management area (www.adfg.alaska.gov/index.cfm?adfg=fishingCommercialByFishery.statmaps) from the fishing start through fish landed dates. A series of additional conditions were required to account for nuances associated with short trips, multiple trips ending on the same day, gaps in VMS transmissions, and trips that offloaded to multiple processors or over multiple days (S3 Text).

#### Calculating trip characteristics and ground-truthing the trip algorithm

To address our second objective, we first calculated the distances traveled and the durations of trips. The majority of trips (88%) had an *in-port* record at both their start and their end, but in most cases, that *in-port* record had a distance from port > 0 nmi (because vessels dock in any number of places within a port). For consistency, if the port was neither Dutch Harbor nor Akutan (which are described below) we linearly extrapolated the vessel’s trajectory to the point at which distance from port was 0 nmi. If an end port was missing however, we had to first resolve missing port information. If a trip was matched to a fish ticket and the trip’s end port was missing due to a gap in the VMS data, the missing port was assigned from the fish ticket port. To ensure the quality of such port assignments, we examined the locations and times of VMS records on either side of the VMS gap and we calculated the speed that would have been necessary for the vessel to have reached the newly assigned port and each of the VMS records on either side of the gap. If that speed was > 14 knots, we instead assigned the final port to be the closest port to the final VMS record prior to the vessel’s entry to port.

The large spatial buffer zones (10 nmi) around Dutch Harbor and Akutan required a different approach to calculating trip durations and distances traveled. Vessels in these ports may have spent substantial amounts of time in transit while still *in-port* and their distances traveled may have been greater than estimated by the 10 nmi port threshold alone. A full analysis of *in-port* behaviors (e.g., ferrying, fueling, delivering) was beyond the scope of our study, but ignoring *in-port* behaviors altogether left the potential for under estimating trip durations and biasing comparison with observer estimates of trip durations, which start at the dock. By analyzing those trips for which contiguous VMS data (≤ 30 min between records) were present between the dock and the 10 nmi threshold, we estimated mean durations and distances traveled by vessels within both Dutch Harbor and Akutan. Vessels were estimated to travel 13 nmi (in 101 min) and 12 nmi (in 92 min) within Akutan at the beginning and end of each trip, respectively. In Dutch Harbor, vessels traveled on average 10 nmi (in 80 min) at both the start and ends of trips. These constants were added to the duration and distances traveled by vessels, starting at the point at which they crossed the 10 nm threshold (see S4 Text for details).

On rare occasions, (< 0.1% of observed trips) a vessel repeatedly crossed the 10 nmi threshold near Dutch Harbor during a short time window, perhaps due to fishing, shuttle runs or gear testing. These would result in inexplicable, repeated trips of very short duration (typically < 4 VMS records, or ~ 120 min). All trips of four or less VMS records total were removed.

Trip distances were calculated by summing the distances between each VMS record plus any of the *in-port* constants or extrapolations to port that were described above. Trip durations were calculated between the first and last VMS record plus any *in-port* constants or extrapolations to port.

Observed and VMS trips were matched for comparison if at least one VMS record fell within the observed trip period and the observed trip duration was > 200 min (73.1% of observed trip records; 0.07% of observed fishing trips). Most of these short observed trips (< 200 min) did not have a matching VMS trip because by our definition they never left port (e.g., a refueling trip within port, moving from the processor to a different dock), and VMS records were often sparse during such periods. Trips of such short duration with VMS data could have measurement errors > 50% and were deemed outside of the precision of our approach. For the remainder of trips, we expected that the VMS-based trip duration may systematically over or under estimate the observer-based trip duration (which we assumed to be the true duration) so we fit a series of regression models to estimate and then, if present, to correct any bias (S5 Text).

### Characterizing fishing versus non-fishing trips

To address our third objective, we used a multi-tiered approach to characterize fishing and non-fishing trips. Our goal was to parse AFA pollock fishing trips from non-fishing trips (e.g., transits between Dutch Harbor and Akutan or working as a tender) and from fishing trips for non-AFA target species or fisheries (e.g., crab or other groundfish). The first step of trip characterization was based on a set of decision rules (S6 and S7 Texts) that examined fish ticket matches, ports, gear types, vessel speeds, trip location and date. The second step relied on regression to predict those trips that still remained uncharacterized after the decision rules. In the final step, we used a set of spatial and behavioral filters to differentiate AFA and non-AFA fishing trips (e.g., if a vessel had no fish tickets for AFA trips within a given month, any unmatched VMS trips during that month were classified as non-AFA trips).

Not all trips could be classified using the decision rules so we fit a regression model to predict fishing versus non-fishing for the remaining, unassigned trips (N = 1,782). We used the already classified trips to fit the model, and among these, we used only those trips that were likely to be representative of the remaining uncharacterized trips (e.g., all trips that were part of scientific surveys or that occurred in certain regions had already been characterized as non-fishing). We also omitted trips for which a single trip overlapped with multiple observed trips, or vice versa, to avoid ambiguous model inputs. Finally, we excluded long (> 15,000 min) and short (<200 min) trips from the training data as they were unrepresentative of the remaining uncharacterized trips.

Binomial generalized linear and additive models (GAMs; R package mgcv version 1.8–4 [21]) were fit to 22,260 already characterized fishing and non-fishing trips to predict the probability that a given trip was a fishing trip. Candidate models were evaluated based on predictive accuracy using training and test datasets of 75% (N = 16,695 trips) and 25% (N = 5,565) of the data, respectively. A suite of trip and vessel characteristics were explored as potential predictors (Table 2), with models iteratively fit via removal of covariates. Smoothing was examined with default selection and with univariate smoothers constrained to 4 estimated degrees of freedom. The final logistic GAM formulation was

$$\text{logit}\left(\text{p}\left(\text{fishing}\right)\right)\;=\;{\text{s}}_{1}\left(\text{ln}\left(duration\right),\;avesp\right)+{\text{s}}_{2}\left(sddif\right)+{\text{s}}_{3}\left(sdsp\right)+\;season_{j}+\;start_{k}+\;end_{l},$$ (1)

where s_*i*_(.) represents the individual smoothing functions. We used an isotropic bivariate smoother [21] for *avesp* and *duration* because longer trips are likely to have more transits and thus, higher average speeds. Univariate smoothing functions (default thin plate regression splines) were fit for the remainder of predictors and the final model used default smoothing.

**Table 2:** Candidate predictor variables for predicting whether a trip is a fishing or non-fishing trip. Trip-level predictors are based on the characteristics of all VMS records per trip that meet the given descriptions.

Candidate predictors	Description	Expectation
avesp	Average speed for all VMS records per trip > 10 nmi from port and traveling > 0 knots	Trips with lower average speeds are more likely to be fishing trips.
duration	Trip duration (min)	Fishing trips are typically 1–4 days
sddif	Standard deviation (per trip) of the difference between speeds of consecutive VMS records when traveling between 0–5 knots (fishing speeds)	Trips with more variability among their slower VMS records are less likely to be engaged in fishing (trawling speeds tend to be fairly constant).
avedif	Average (per trip) of the difference between speeds of consecutive VMS records when traveling between 0–5 knots (fishing speeds)	Trips with very slow (< ~ 1 knots) average speeds among their slower VMS records are less likely to be engaged in fishing.
sdsp	Standard deviation of speed for VMS records per trip > 10 nmi from port and traveling > 0 knots	Trips with a higher variability of speed are more likely to be fishing.
avgspstat	Average speed for VMS records per trip occurring in statistical management areas known as “fishing areas”	The average speed at fishing grounds is likely to be slower if fishing occurs.
start_k_	Port from which the trip began, grouped into one of four regions: Gulf of Alaska, Bering Sea, Aleutian Islands, or Other (see S6 Text for breakdowns by port)	Fishing trips are less likely to occur if started from certain ports.
end_l_	Port in which the trip ended, grouped into same regions as in start_k_	Fishing trips are less likely to return to certain ports.
season_j_	Pollock fishing is divided into a winter “A” season and a summer “B” season, with “N” representing non-pollock season trips.	Vessel often target different locations during the different seasons, which would affect statistical moments calculated for speed.
size	Vessel length	Smaller vessels may transit and fish differently than larger vessels.

### Bias estimation from simulation of VMS gaps

The primary complication with the use of VMS data was inconsistent transmission intervals. In addition to complicating the trip algorithm, gaps in VMS transmissions may have also affected the estimated distances traveled. The calculated distance traveled will depend on the trajectory of the vessel between VMS records. Inevitably, data that are sampled as infrequently as VMS data (as opposed to AIS data, which are collected constantly) will under-estimate distances traveled (and subsequently, vessel speeds), because we calculate only straight-line distances between VMS records. The role of temporal sampling resolution on inference is the subject of entire papers (e.g., [22–24]) and thus a full assessment of errors in the estimated distance traveled was beyond the scope of this study. However, previous studies primarily focused on mandated transmission frequencies (e.g., all VMS records being transmitted at 30 min vs. 60 min intervals). These studies did not address the role of missing data or gaps so we conducted a basic simulation to demonstrate how a single or several missed VMS records may affect the travel distance for a given trip.

We simulated gaps in VMS transmission > 30 min by removing VMS records from trips with complete data sets. We removed a single record from a trip such that the VMS data would have a single gap of 60 min instead of 30 min. Removal of a second VMS record (adjacent to the first) would yield a gap of 90 min, and so on, for additional removals. To simulate the effect of such gaps, we identified trips whose VMS records were transmitted at regular intervals (25–35 min) and we removed 1–4 consecutive VMS records from each trip to simulate gaps of 60, 90, 120, and 150 min. We randomly sampled 5,000 trips, with replacement. Distances traveled between consecutive records varied with vessel behavior throughout the course of a trip, so the position of the removed VMS record within the trip sequence was also randomly chosen (thus, sampling with replacement was not a concern). The subsequent removals occurred at the positions one, two, or three VMS records prior to the first removal.

## Results

Our first objective was to use VMS data to identify individual trips made by CVs in the BSAI and GOA. We expected this objective to be a straightforward “vessels leave port and then return to port” analysis but as many VMS records (8.9%) were transmitted at 35–60 min intervals (instead of the expected 30 min) and another fraction (2.1%) were transmitted less frequently, our method evolved into the more intricate presentation described above. This development however underscores the value of understanding the data at hand and emphasizes that even though software packages exist for processing VMS data, it is still valuable to understand how the particular dataset may affect inference. By scrutinizing the data, we developed an algorithm to accommodate variable transmission rates and we identified 29,969 trips from 2008–2014, of which 15,418 were matched for comparison with observed trips.

### Calculating trip characteristics and ground-truthing the trip algorithm

**Fig 1:**
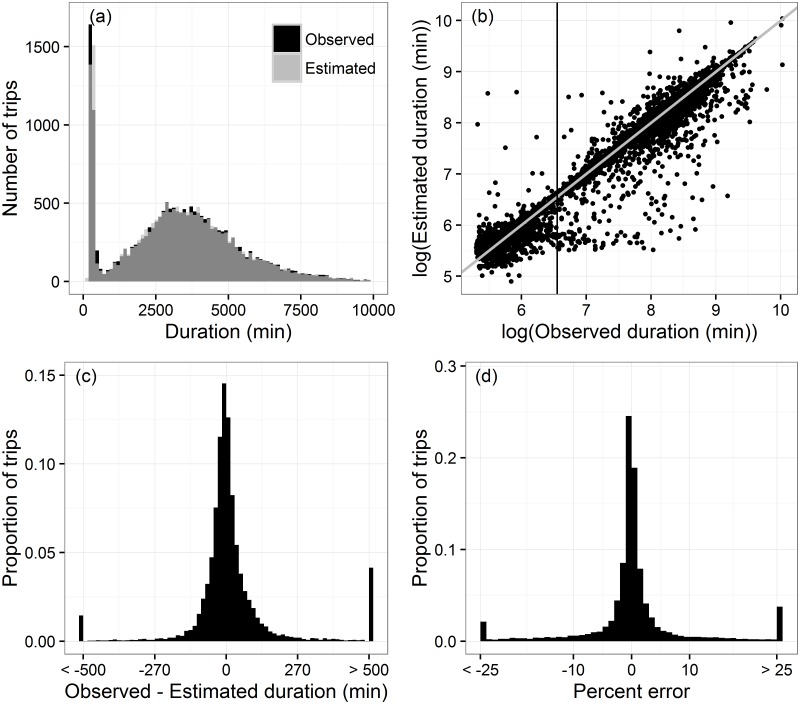
Distributions of observed and VMS-estimated trip durations. (a) Overlain histograms illustrating the distribution of durations for observed and VMS-estimated trips. Dark grey areas show overlap between the two histograms. For illustration purposes, figures are scaled to a maximum of 10,000 min which omits < 1% of trips with longer durations. (b) Scatterplot of the observed versus VMS-estimated duration for each trip. Data are log-transformed to better illustrate the clusters of data greater than and less than ~ 700 min. The vertical line shows the log-transformation of 700 min (6.55), the cutoff for exploring different models to estimate bias in duration estimation. The grey line represents the 1:1 line. (c) Histogram of the difference between the observed and estimated duration for each trip. For illustration purposes, values less than -500 and values greater than 500 have been grouped into single bins, “< -500” and “>500,” respectively. (d) Histogram of percent error (positive errors indicate over-estimation) of observed versus estimated trip durations. For illustration purposes, values less than < -25% and greater than 25% have been grouped into single bins, “< -25” and “> 25.”

Our second objective was to quantify the distances and durations of each trip and to ground-truth the estimated trip durations with observed trip durations. VMS and observed trips were matched when at least one VMS record fell within the observed trip period. A VMS trip that started and ended at exactly the same time as an observed trip would have no difference in the estimated versus observed duration. However, even cases where the trip algorithm perfectly captured the dynamics of a trip still have a range of expected error. For example, if VMS records were transmitted at 12:00 and 12:30, and an observer reported a trip to start at 12:01, the algorithm would start the trip at 12:30 and a 29 min difference would exist between the VMS and observed trips. Similarly, if the observer ended a trip at 12:01 but the next VMS record did not appear until 12:30, a 29 min difference in trip endings could exist. Thus, even with VMS records transmitted at regular 30 min intervals, the difference could be as large as 58 min (29 min at the start and 29 min at the end of a trip). This measurement error would increase as the time between VMS records increased, such that the error would be two times the VMS transmission rate minus two minutes. The duration of a trip impacts the significance of this measurement error. For example, for trips longer than 1,160 min (19.3 hrs), the 58 min measurement error represents < 5% of the total trip. Among observed trips (Fig 1), 86.4% of durations were estimated within their measurement error, 76% of estimates were ≤ 5% of the observed duration, and 96.5% of durations were estimated either within their measurement error or within 5% of the observed duration. Estimated and observed trip durations had a Spearman ρ = 0.98.

The distribution of trip durations was bimodal (Fig 1a), with predominantly non-fishing trips < 700 min and fishing trips ≥ 700 min. As the 1:1 line (Fig 1b) illustrates, trips ≥ 700 min were both under and over estimated while trips < 700 min appeared to be biased toward over-estimation for the shortest trips (more points above the 1:1 line) and under-estimation as trips got longer (more points below the 1:1 line). The aggregate duration (sum of all durations) of VMS trips was 1.4% less than the aggregate duration of observed trips and trip-level durations had a mean absolute error of 5.78%. Regressions to correct for bias in estimated trip duration yielded mixed results, with aggregate durations slightly improved by models and trip-level durations slightly worsened (see S5 Text for regression details; this approach may be quite effective in other fisheries or regions). These findings underscore that multiple types of behaviors may exist within a given fishing fleet. While a regression-based approach to correct biases in our study did not have a large effect, it may have more traction in different fisheries and is a valuable tool to have at the analyst’s disposal.

Only a small fraction of trips (~0.1%) had absolute errors greater than 100%. All of these were the result of over-estimated trip durations, occurring because the algorithm was unable to detect the transition from one trip to the next. The majority of these resulted from gaps in VMS data greater than 4 hours while the remainder were a combination of cases where the trip algorithm simply missed a trip transition (e.g., the speed conditions around a particular port did not trigger a new trip), a floating processor was too far from the GPS coordinate we used to define it as a “port,” or the observer-reported time did not align with the vessel’s return/ departure from port. The small error rate suggests that the trip algorithm approach worked well and that regardless of how well the algorithm is tuned, there will inevitably be data issues that will result in at least small amounts of error.

### Classification of trips

Decision rules classified 19,877 trips as either fishing or non-fishing. Among those classified trips with matching observer data, 99.9% (N = 11,678) and 98.8% (N = 2,777) of observed fishing and non-fishing trips, respectively, were correctly assigned.

The GAM explained 91.1% of the model deviance and demonstrated a 99.0% accuracy predicting out-of-sample (N = 1,210 non-fishing trips, 4,355 fishing trips). The model predicted whether fishing occurred during the remaining 1,768 unclassified trips. Among these trips, 386 and 1,382 were designated as non-fishing and fishing, respectively.

The combination of decision rules and regressions classified 29,794 trips (99.4%) as fishing or non-fishing, and as either an AFA pollock trip or a non-AFA fishing trip (Table 3). The final distribution of non-fishing, non-AFA fishing trips and AFA fishing trips by year (0.6% of trips remained unclassified and are omitted here) is shown in Table 3 (see S8 Text for more detailed descriptions of trip type compositions).

**Table 3:** Distribution of fishing and non-fishing trips. Total numbers of trips and vessels, plus the percent of the total trips for AFA fishing, non-AFA fishing, and non-fishing trips. Annual tallies are provided by season (Winter “A” season, Summer “B” season, and “N” non-AFA season).

Season	Year	Total trips	AFA trips	Non-AFA trips	Non-fishing trips	Vessels with AFA trips	Vessels with non-AFA trips
A	2008	1459	40.7	42.2	17.1	75	61
A	2009	811	45.4	35.6	19	66	37
A	2010	1394	39.1	37.4	23.5	76	51
A	2011	1873	43.4	34.2	22.4	80	52
A	2012	1904	41.7	36.6	21.7	80	55
A	2013	1765	42.6	36	21.4	73	52
A	2014	1710	43	40	17	68	53
B	2008	2069	51.9	14.5	33.6	74	40
B	2009	1668	44.9	13.2	41.9	69	31
B	2010	2140	39.8	12.1	48.2	69	27
B	2011	2896	45.1	10.5	44.4	74	33
B	2012	2538	49.9	11.3	38.8	76	34
B	2013	2788	43.1	11.3	45.6	72	38
B	2014	2297	50.9	8	41.1	73	31
N	2008	467	0	50.7	49.3	0	57
N	2009	649	0	59.8	40.2	0	77
N	2010	326	0	28.5	71.5	0	27
N	2011	137	0	19.7	80.3	0	13
N	2012	165	0	19.4	80.6	0	12
N	2013	290	0	34.1	65.9	0	28
N	2014	515	0	56.1	43.9	0	40

As demonstrated by the above percentages, our combination of decision rules and regressions yielded highly successful classifications of trips, when compared with observer data, supporting our objective of classifying trips as non-fishing, fishing for pollock, or fishing for species other than pollock, based on trip-level characteristics like gear type, port, and average speeds. Furthermore, by laying out the rationale behind the chosen model covariates (Table 2) we believe that adjusting this approach for other fisheries and with other data should be relatively straightforward, even if different decision rules and different final models are ultimately necessary.

### Bias estimation from simulation of VMS gaps

**Fig 2:**
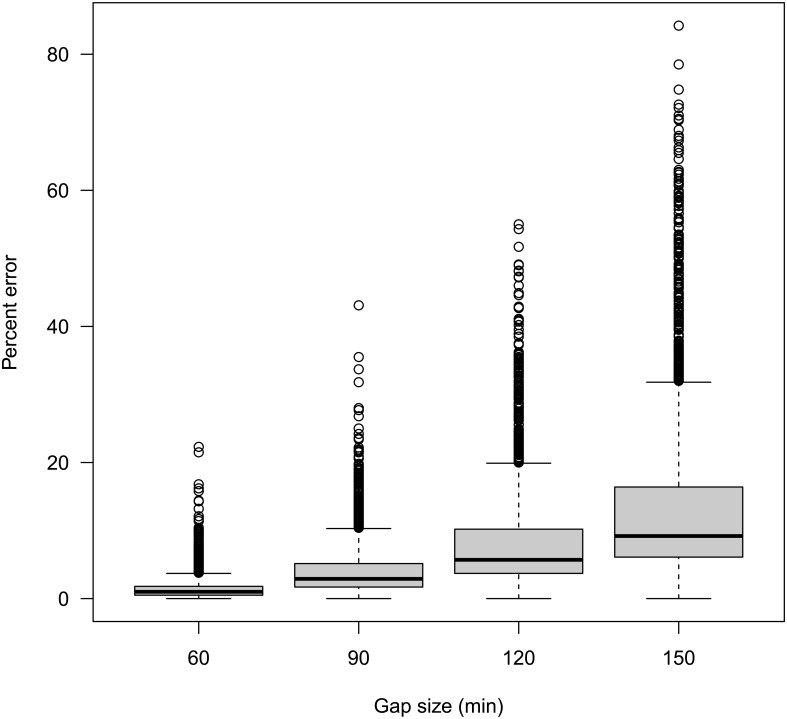
Percent errors in the estimated trip duration as a function of time gaps in VMS transmissions. Gaps in the regular transmission frequency greater than the expected 30 min intervals were simulated by removing 1–4 VMS observations from a random location within a trip’s sequence of VMS records. Removals yielded gaps in the VMS sequence of 60, 90, 120, and 150 min. Points outside of the whiskers represent outliers (> 1.5 times the upper quartile), whiskers represent the range (excluding outliers) and the boxes represent upper and lower quartiles with the median depicted by the horizontal line within each box.

Understanding how variability in the transmission of data may affect inference is critical for evaluating the utility of data and any approach that uses those data. While other studies have examined the roles of sampling frequencies, we have simulated the role that data quality (in the form of discontinuities in the data) may affect conclusions. Simulations that removed one, two, three, and four consecutive VMS records from a trip (Fig 2) reduced estimated trip distances (compared to the same trips without gaps) by a mean (± 1 mean absolute deviation [MAD]) of 1.5% (± 1.1%), 4.1% (± 2.6%), 8.1% (± 5.0%), and 13.4% (± 8.3%), respectively. While only a relatively small portion of trips had appreciable gaps in VMS data, these gaps are capable of substantially reducing trip distances. However, given the high degree of accuracy seen when estimated trip durations were compared with observed trip durations, we believe that the effects on trip distances were insufficient to invalidate our methods. They may however provide guidance on estimating error rates when extending such analyses to fuel consumption calculations or other trip-level metrics.

## Discussion

Many studies have used VMS to examine facets of fishing behavior, often analyzing copious amounts of information with little discussion of the nuances of data processing and management or how necessary assumptions may affect interpretations. We have examined such subtleties through providing a complicated answer to the trivial questions, “When does a fishing trip start and end?” and “What type of fishing trip was it?” As we demonstrated, nuanced approaches were required to account for region-specific aspects of the data (e.g., geography of fishing ports and targeting behavior) and discrepancies between different data sources (e.g., inconsistent VMS transmissions and imprecise dates/ times in human-recorded data). The challenges that we have addressed here—while specific in their geography and peculiarities—represent several of the key challenges facing users of VMS data globally. These challenges represent some of the critical road blocks that researchers and managers face when using VMS data to resolve metrics like trip-level effort, trends in fleet behavior, responses of the fleet to climatic or regulatory changes, and dynamic costs to fishers. While much of the subsequent discussion describes the important challenges to using these “big data,” we emphasize that highly accurate results were still obtainable by taking extra steps to understand and account for data issues.

### VMS Data

Spatial data are increasingly available to track the movements of marine vessels worldwide. Fishery researchers have recently found two such sources of data to help resolve unobserved fishing behaviors globally. Automatic identification systems (AIS) and VMS rely on historically different technologies and purposes. Designed to improve maritime safety, AIS transmit high resolution location data (typically < 1 second intervals) from vessels in real-time and they inform neighboring vessels of their movements. Traditionally, AIS was based on VHF transmissions and required line-of-sight to another vessel or a shore-based receiver in order to transmit data. However, more recently, satellite AIS systems have enabled tracking of vessel movements across the globe. Given the virtually continuous streaming of AIS data, this technology offers promise for improving prediction of vessel behaviors (e.g., [25]) and correcting some of the biases introduced by the relatively longer sampling frequencies of VMS data. However, proximity to shore-based receivers and poor satellite reception can affect data quality (e.g., in the North Pacific) [26], and exemptions have historically existed for some fishing vessels, allowing them to deactivate their AIS units to protect the confidentiality of fishing grounds. At present, AIS data access can also be difficult (we tried to obtain AIS data for this study) so while improving technology offers an alternative or a complement to VMS data, some challenges still remain. Meanwhile, VMS data offered much potential, despite having challenges of their own.

The irregular and sometimes long gaps in VMS transmissions were responsible for the majority of the challenges encountered in this study. Gaps may occur when vessels receive permission to deactivate their VMS units, have equipment failures or poor satellite reception, or are a result of illicit behavior / tampering. Gaps in coverage are scarcely mentioned in the VMS literature (though see [6,27]), but preliminary exploration of VMS data from a different region of the United States also found gaps, suggesting that they may be pervasive and thus, critical to how VMS data are analyzed. Without such gaps, our approach would have been dramatically simplified; transitions into and out of ports would have been more readily captured and a simple point-in-polygon approach like that of existing software (VMStools [13] and VMSbase [14]) would have likely been sufficient. Instead, > 10% of trips were missing an *in-port* VMS record at the beginning or the end of the trip, thereby precluding an easily identifiable trip start and end. Meanwhile, some trip transitions were missed altogether because gaps spanned the entire *in-port* period. These missing port periods required a more substantial approach to trip identification, but after careful accounting, comparison with observed trip durations (average errors < 1.5%) suggested that some of the data complications were well-accounted for.

**Fig 3:**
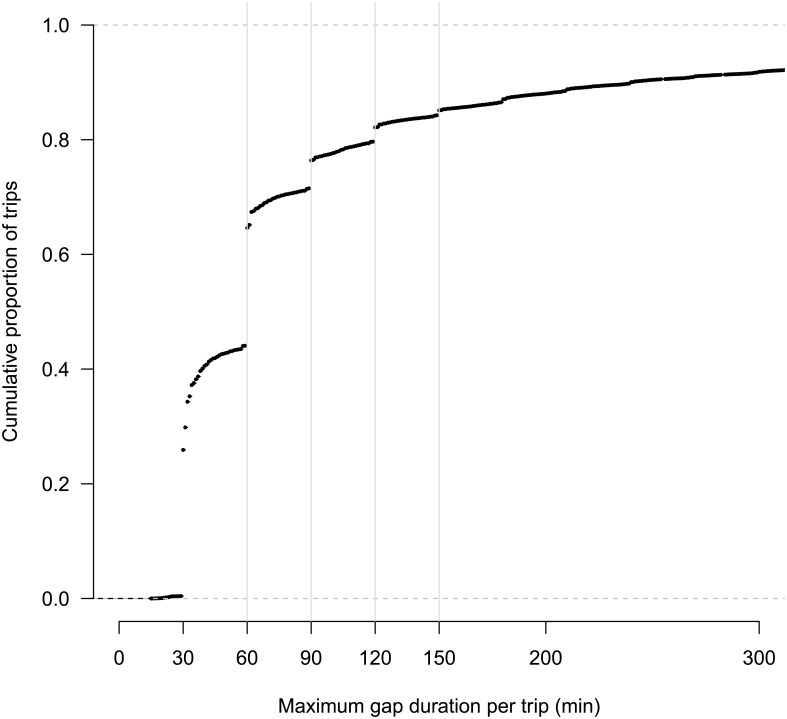
Cumulative distribution of the maximum time gap between VMS records for each trip. For illustration purposes, the 10% of trips with maximum gaps > 300 min are not shown here. Vertical grey lines are shown at each of the gap durations for which we simulated removals of VMS records.

Even when gaps in VMS transmissions did not affect trip identification, the missing information associated with the gaps was still important. Simulated gaps yielded mean underestimation of trip distances (± 1 MAD) of 1.5 (1.1) % to 13.4 (8.3) %. These results were particularly poignant given that ~ 34% of actual trips had at least one gap > 60 min and ~15% of trips had at least one gap > 150 min (Fig 3). While the implications of this are straightforward—gaps in VMS data often lead to underestimation of trip distance—the range of the errors (Fig 2) is also important. In each of the gap scenarios, the lower end of the range was zero percent difference. This highlights the importance of the vessel behavior when a gap occurs. If a vessel is transiting in a straight line for the duration of the gap, little or no error may occur in the estimation of distance. However, as the sinuosity of a vessel’s path increases, especially at higher speeds, the error in travel distance will increase. A substantial body of literature has examined the role that different VMS transmission frequencies may play on the estimation of effort [11, 22, 23] and several studies [28–29] have presented interpolation techniques for resolving coarse temporal sampling in vessel tracks. However, while such studies provide valuable discussions of broader errors associated with periodically sampled data, they focus on systemic biases instead of the smaller yet still substantial errors that may be introduced and easily overlooked by a single or only a few missing VMS records. We hope that despite the simplicity of the simulations presented here, users of VMS data will recognize the dramatic impact that even small gaps in VMS transmissions have on inference.

### Human-recorded data (“the truth”)

We used observer data from more than 15,000 trips as the empirical information with which we performed validation. However, in some cases, inscription errors may exist in the observer data themselves, resulting in failed matches with VMS trips, or more frequently, leading to under or over estimation of duration as compared to the VMS data. Two typical cases emerged upon manual inspection of many matched trips. Observed trips are defined as starting when a vessel unties from the dock and ending when it ties up at the dock again. However, it was not uncommon for the observed start of a trip to occur while the vessel remained at the dock (or vice versa, at the end of the trip), sometimes for extended periods. While these at-the-dock periods led to erroneously long trips, other situations occurred where the vessel was several miles and/ or hours outside of port when the observed trip began or ended, leading to observed trip durations that were shorter than the apparent trip duration based on VMS. Notes in observer logs may explain such exceptions, but notes may be infeasible to incorporate with datasets of this size. Nonetheless, cases like these are responsible for discrepancies between the observed and VMS trip durations that are unrepresentative of the true errors of our approach.

Fish tickets were the other human-recorded data source upon which we relied. They were used for identifying missing port information, as well as identifying fishing and non-fishing trips and parsing AFA from non-AFA fishing trips. Several aspects of the fish ticket data complicated this matching procedure. However, the matching of fish tickets relied on the dates that were manually recorded, so incorrect or imprecise dates could lead to errors in the matching or an inability to match trips altogether. For example, VMS could identify that a vessel that reported landing its catch in Dutch Harbor on January 20^th^ did not come within 30 nmi of Dutch Harbor until January 21^st^. Alternatively, a vessel may have delivered their catch on January 20^th^ but the fish ticket reports January 21^st^, when the vessel is already 30 nmi from port. In our case, many fish tickets were omitted from matching because the dates were clearly incorrect but the correct date was unclear.

While recording, rounding, transcription, or other time-keeping differences may have led to errors in matching VMS trips with observer data or fish tickets, or may have led to errors in their comparisons, manual inspection of many matched trips suggested that these human-reported errors were relatively rare in the NPGOP data. This was not surprising given the scale of the NPGOP and the several decades of development they have had for quality control protocols. However, our approach was able to identify errors and it may offer observer and fish ticket programs globally an additional method by which to assure the quality of their datasets. For example, we could modify this algorithm so that the observer program could examine cases where the VMS trip duration was misaligned with that of the observed trip duration and in plotting the vessel speeds and distances from port, they could easily identify—within 30 min—when the vessel actually left or returned to/ from port. Similarly, one example of an error in fish ticket data occurred when a fish ticket reported having a fishing start date of 01/03 and an end date of 02/01. However, there were three other fish tickets for this vessel occurring on 1/20-1/22, 1/23-1/24, and 01/25-01/27. In such a case, it is was clear that either the start or the end date of the fish ticket was wrong, but without knowing where the vessel was during either of those dates, it would be impossible to rectify the error. By mapping the VMS trips that occurred during that period, it was trivial to rectify which of the two dates was the incorrect one. Similarly, by identifying all of the fish tickets matched to a single VMS trip, errors in reporting might be identified or fish tickets might be more effectively grouped for other analyses. Finally, data fields in fish tickets were occasionally blank or port locations were recorded incorrectly, and these could be rectified via VMS-based vessel location.

### The trip algorithm

Our algorithmic approach performed well, matching observed trips and estimating their durations with differences typically < 1.5%. Some of the discrepancies that did occur between observed and estimated trips were the result of different trip definitions between the algorithm and observers. For example, if a vessel anchored in port instead of tying up at the dock, the algorithm ended the trip (such differences were indistinguishable by VMS) but the observer did not. Similarly, some stops at floating processors were identified as new trips by the algorithm but not by the observer. Other rare discrepancies occurred when the algorithm missed the transition between observed trips; even with regular VMS transmissions, brief stops that occurred between VMS records occasionally remained undetected. An additional point of discrepancy could occur if a vessel spent longer than average within the port boundary for Dutch Harbor or Akutan. For example, a lineup at the fuel dock or a long wait to deliver to a processor could result in greater than expected durations from observed trips such that the *in-port* constant added to trip durations was low. In other rare cases, floating processors were in unexpected locations and were not among the list of port coordinates in the algorithm so trip transitions were missed altogether. Finally, a transiting vessel may have passed close enough to a port while also satisfying the algorithm’s speed conditions for that port, such that a new trip was incorrectly triggered by the algorithm. Our algorithm was finely tuned to account for as many of the above contingencies as possible while recognizing that without over-fitting, higher accuracy was unlikely. Nonetheless, some vessel movements and transitions were simply unable to be captured or anticipated. However, the majority of the errors that did occur were more likely the result of data issues than model fits.

### Applications and future directions

Despite numerous data challenges, VMS provide a method by which trip characteristics are estimated to a high degree of accuracy. We further identify types of vessel and targeting behaviors, making this study the first, to our knowledge, to use VMS to identify métiers [30] (specific fisheries by gear, region, target species) in U.S. fisheries. In the Bering Sea, all trips targeting pollock are now observed but our approach enables us to characterize unobserved trips elsewhere throughout the North Pacific and retrospectively for years prior to full observer coverage. Our continuing steps include applying our approach (developed with 91 trawl vessels) to more than 500 longline and trawl vessels with VMS in the BSAI and GOA. Meanwhile our approach has been adapted for vessels in the Gulf of Mexico (using longlines, troll gear, trawl, hand lines, pots, traps, and divers) indicating the extent of the generalizability. Our approach may help other VMS users in the future to quickly identify the role that gaps may play in their dataset, as well as how geography of their particular ports may affect inference.

Contemporary fisheries literature is rife with projections of climate-induced shifts in fish populations and the subsequent implications for global fisheries (e.g., [31] and references therein). Range expansion of fish populations [32–34] may be accommodated by longer transits to fishing grounds or by increased search time between fishing activities. However, even if catch per unit effort, as typically defined based on active fishing time, can be maintained under longer transit scenarios, costs to fishers may increase [1], and ultimately, the profitability (and thus, sustainability) of fisheries may be compromised. Precise estimates of travel distances, trip durations, and the diversification of fishing strategies (e.g., change in métiers per vessel over time) [35] may thus become a critical component to understanding and characterizing the resilience of certain fisheries. For example, a shift in pollock populations away from port would impact the smaller vessels in the fleet more than the larger vessels which have greater hold capacity and a greater ability to buffer against increased fuel costs [36]. Linking trip characteristics with extrinsic factors like fuel price allows analyses to estimate the breaking points at which vessels change their fishing behavior and ultimately alter their impacts to the coastal economies that are supported by them.

This study presents a methodology for assessing trip characteristics when pre-packaged software (e.g., VMStools, VMSbase) are incompatible (e.g., due to missing port information or port geography outside of the programmed regions) with a particular dataset or level of precision. Additionally, as we discovered, even a dataset with coverage of the entire fleet for more than a decade is likely to require greater than expected scrutiny. That scrutiny may increase as additional datasets (e.g., observer, fish ticket or logbook data) are brought into the mix, while also presenting new avenues for quality control across data programs.

## Supporting Information

S1 Text Calculation/ description of fields from VMS data. Consecutive VMS records were used to calculate vessel distances from port, VMS transmission intervals, and vessel speed. Shapefiles were used for matching VMS records to State and Federal management areas.
(DOCX)

S2 Text Trip algorithm conditions for *in-port* status. A series of conditional statements including vessel speeds, distances from port, and time between VMS transmissions were required for determining whether vessels were *in-port*. Port-specific conditions are detailed here.
(DOCX)

S3 Text Matching fish ticket data to VMS records. Fish tickets and VMS records do not contain timestamps on the same scale and fish tickets dates are manually recorded and can involve complicated multi-day or multi-port deliveries. We detail the matching of these two datasets here.
(DOCX)

S4 Text Calculation of in/near port distances. Time and distances traveled while vessels are *in-port* can affect the comparison between observed and VMS-based trip durations. Here we describe the process of identifying offset values to account for such behaviors.
(DOCX)

S5 Text Regression to correct estimated trip duration. We explore regressions to estimate and correct for bias in the estimation of trip duration as compared to observer data.
(DOCX)

S6 Text Decision rules for characterizing types of trips. We describe the conditions used to identify trips as fishing or non-fishing and for those trips identified as fishing, we further assign them as fishing for pollock in the Bering Sea or having other targeting behavior.
(DOCX)

S7 Text Determination of non-fishing corridors near Dutch Harbor. We describe the process of spatial matching that identifies non-fishing transit corridors between the ports of Dutch Harbor and Akutan and between Dutch Harbor and a floating processor.
(DOCX)

S8 Text Distribution of fishing and non-fishing trip types and ports. We present the distribution of trip types and ports as a complement to Table 2.
(DOCX)

## References

[1] Haynie AC, Layton DF. An expected profit model for monetizing fishing location choices. J Env Econ Manage. 2010;59: 165–176.

[2] Haynie AC, Pfeiffer L. Climatic and economic drivers of the Bering Sea walleye pollock (*Theragra chalcogramma*) fishery: implications for the future. Can J Fish Aquat Sci. 2013;70: 841–853.

[3] Joo R, Bertrand A, Bouchon M, Chaigneau A, Demarcq H, Tam J, et al Ecosystem scenarios shape fishermen spatial behavior. The case of the Peruvian anchovy fishery in the Northern Humboldt Current System. Prog. Oceanogr. 2014; 128; 60–73.

[4] Abernethy K E, Trebilcock P, Kebede B, Allison E H, Dulvy N K. Fuelling the decline in UK fishing communities? ICES J Mar Sci. 2010; 67:1076–1085.

[5] AFSC (Alaska Fisheries Science Center) Observer Sampling Manual. Fisheries Monitoring and Analysis Division, North Pacific Groundfish Observer Program. AFSC, 7600 Sand Point Way N.E., Seattle, Washington, 98115. 2016

[6] Chang S, Yuan T. Deriving high-resolution spatiotemporal fishing effort of large-scale longline fishery from vessel monitoring system (VMS) data and validated by observer data. Can J Fish Aquat Sci. 2014;1370: 1363–1370.

[7] Lee J, South AB, Jennings S. Developing reliable, repeatable, and accessible methods to provide high-resolution estimates of fishing-effort distributions from vessel monitoring system data. ICES J Mar Sci. 2010;67: 1260–1271.

[8] Bastardie F, Nielsen JR, Ulrich C, Egekvist J, Degel H Detailed mapping of fishing effort and landings by coupling fishing logbooks with satellite-recorded vessel geo-location. Fish Res. 2010;106: 41–53.

[9] Palmer MC, Wigley SE. Using positional data from vessel monitoring systems to validate the logbook-reported area fished and the stock allocation of commercial fisheries Landings. Nor Amer J Fish Man. 2009;29: 928–942.

[10] Jennings S, Lee J. Defining fishing grounds with vessel monitoring system data. ICES J Mar Sci. 2012; 69: 51–63.

[11] Mills C M., Townsend SE, Jennings S, Eastwood PD, Houghton CA. Estimating high resolution trawl fishing effort from satellite-derived vessel monitoring system data. ICES J Mar Sci. 2007;64: 248–255.

[12] Stelzenmüller V, Rogers SI, Mills CM. Spatio-temporal patterns of fishing pressure on UK marine landscapes, and their implications for spatial planning and management. ICES J Mar Sci. 2008;65: 1081–1091.

[13] Hintzen NT, Bastardie F, Beare D, Piet GJ, Ulrich C, Deporte N, Egekvist J, Degel H. VMStools: Open-source software for the processing, analysis and visualisation of fisheries logbook and VMS data. Fish Res. 2012;115–116:31–43.

[14] Russo T, D'Andrea L, Parisi A, Cataudella S. VMSbase: An R-Package for VMS and Logbook Data Management and Analysis in Fisheries Ecology. PLoS ONE 2014;9(6): e100195 10.1371/journal.pone.0100195 24932915 PMC4059747

[15] Murawski SA, Wigley SE, Fogarty MJ, Rago PJ, Mountain DG. Effort distribution and catch patterns adjacent to temperate MPAs. ICES J. Mar. Sci. 2005; 62: 1150–1167.

[16] Fissel B, Dalton M, Felthoven R, Garber-Yonts B, Haynie AC, Himes-Cornell A, et al Stock assessment and fishery evaluation report for the groundfish fisheries of the Gulf of Alaska and Bering Sea/Aleutian Islands area: Economic status of the groundfish fisheries off Alaska. Economic and Social Sciences Research Program, Alaska Fisheries Science Center, National Marine Fisheries Service, National Oceanic and Atmospheric Administration 2015

[17] R Core Team R: A Language and Environment for Statistical Computing. R Foundation for Statistical Computing. http://www.R-project.org. 2015

[18] Spalding, K. NMFS VMS Program Headquarters, 1315 East West Highway, Silver Spring, MD 20910. 2016

[19] NOAA Alaska Fisheries Science Center. North Pacific (NORPAC) Groundfish and Halibut Observer Data Dictionary December 2007 –Present. 2016a. Metadata and access details available at: https://inport.nmfs.noaa.gov/inport/item/7290.

[20] Alaska Department of Fish and Game, and (CFEC) Alaska Commercial Fisheries Entry Commission. 2015. Alaska fish ticket data. Data compiled by Alaska Fisheries Information Network in COMPREHENSIVE_FT. Metadata: http://www.akfin.org/data/documentation/

[21] Wood SN. Generalized additive models: an introduction with R. Boca Raton, FL: CRC/ Chapman and Hall; 2006.

[22] Deng R., Dichmont C, Milton D, Haywood M, Vance D, Hall N, Die D. Can vessel monitoring system data also be used to study trawling intensity and population depletion? The example of Australia’s northern prawn fishery. Can J Fish Aquat Sci. 2005;62: 611–622.

[23] Palmer MC. Calculation of distance traveled by fishing vessels using GPS positional data: A theoretical evaluation of the sources of error. Fish Res. 2008;89: 57–64.

[24] Postlethwaite CM, Dennis TE. Effects of temporal resolution on an inferential model of animal movement. PLoS One. 2013;8(5), e57640 10.1371/journal.pone.0057640 23671558 PMC3646004

[25] Last P, Bahlke C, Hering-Bertram M, Linsen L. Comprehensive Analysis of Automatic Identification System (AIS) Data in Regard to Vessel Movement Prediction. The Journal of Navigation. 2014;67: 791–809.

[26] Renner M, Kuletz KJ. A spatial-seasonal analysis of the oiling risk from shipping traffic to seabirds in the Aleutian Archipelago. Marine Pollution Bulletin. 2015; 101: 127–136. 10.1016/j.marpolbul.2015.11.007 26602441

[27] Joo R, Bertrand S, Tam J, Fablet R. Hidden Markov models: the best models for forager movements? PloS One. 2013;8(8): e71246 10.1371/journal.pone.0071246 24058400 PMC3751962

[28] Hintzen NT, Piet GJ, Brunel T. Improved estimation of trawling tracks using cubic Hermite spline interpolation of position registration data. Fish Res. 2010;101: 108–115.

[29] Russo T, Parisi A, Cataudella S. New insights in interpolating fishing tracks from VMS data for different métiers. Fish Res. 2011;108: 184–194.

[30] Marchal P. A comparative analysis of métiers and catch profiles for some French demersal and pelagic fleets. ICES J Mar Sci 2008;65: 674–686.

[31] Hollowed AB., Barange M, Beamish RJ, et al Projected impacts of climate change on marine fish and fisheries. ICES J Mar Sci. 2013; 70:1023–1037.

[32] Drinkwater KF. The response of Atlantic cod (*Gadus morhua*) to future climate change. ICES J Mar Sci. 2005; 62:1327–1337.

[33] Kotwicki S, Lauth RR. Detecting temporal trends and environmentally-driven changes in the spatial distribution of bottom fishes and crabs on the eastern Bering Sea shelf. Deep-Sea Res II. 2013; 94:231–243.

[34] Nye JA, Link JS, Hare JA, Overholtz WJ. Changing spatial distribution of fish stocks in relation to climate and population size on the Northeast United States continental shelf. Mar Ecol Prog Ser. 2009; 393:111–129.

[35] Kasperski S, Holland DS. Income diversification and risk for fishermen. Proc Nat Acad Sci. 2013; 110:2076–81. 10.1073/pnas.1212278110 23341621 PMC3568353

[36] Criddle K, Strong J. Dysfunction by design: Consequences of limitations on transferability of catch shares in the Alaska pollock fishery. Mar Policy. 2013; 40:91–99.

