# Supplementary material for: Using Vessel Monitoring System Data to Identify and Characterize Trips Made by Fishing Vessels in the United States North Pacific
Source: PLoS One. 2016 Oct 27;11(10):e0165173. doi: 10.1371/journal.pone.0165173 (PMC5082895; doi:10.1371/journal.pone.0165173)
Supplement: S1 Text — Consecutive VMS records were used to calculate vessel distances from port, VMS transmission intervals, and vessel speed. Shapefiles were used for matching VMS records to State and Federal management areas. (DOCX) [file pone.0165173.s001.docx]

**S1_Appendix**

**Calculation/ description of fields from VMS data**

*Distance from port -* The distance of each VMS record from port was calculated using the Haversine formula by iteratively identifying the closest port from among all potential northeast Pacific commercial fishing ports.

*Vessel speed* - Some VMS instruments transmit a vessel’s instantaneous speed but this field was inconsistent in the data for different VMS instruments and during different years so vessel speeds were instead calculated from the time difference and the distance traveled between VMS records (using the Haversine formula for calculating great-circle distances). In most literature studies with tagging or movement data, speeds are either calculated between the current and previous or the current and subsequent records. However, with the relatively long duration between VMS records, we also calculated and utilized the mean of the forward- and backward-calculated speeds. Unless otherwise stated, any reference to vessel speed refers to this average speed term.

*ADF&G and* *NMFS management areas -* VMS locations were linked to a polygon shapefile (via PBSmapping version 2.67.60 in R Statistical Software Version 3.1.1) consisting of 1807 Alaska Department of Fish and Game statistical areas (www.adfg.alaska.gov/index.cfm?adfg=fishingCommercialByFishery.statmaps) nested within 26 NMFS management areas that define fishery boundaries in the BSAI and GOA. It is not unusual for VMS instruments to transmit locations that are clearly erroneous and occur on-land; such records are referenced in other VMS studies (e.g., Hintzen et al., 2012; Russo et al. 2014) and are typically removed indiscriminately. However, in contrast to other studies, VMS records near port were critical to our objectives and some of the seemingly erroneous records were only barely on-land and appeared to be legitimate *in-port* records. These apparent errors may have been the result of different geodetic datums, VMS measurement error, shapefile resolution, extreme tides or another unforeseen factor. The coastline defines the boundary for each of the NMFS management areas and thus, on-land points were not automatically assigned to a NMFS area. Instead of haphazardly removing these points we first determined the distance of each unmatched point to the nearest management area (using the gDistance function in rgeos Version 0.3-8 for R) and if an area was within 5 km (km were used instead of nm because it was the native output from gDistance), the point was matched to that area.

References

Hintzen NT, Bastardie F, Beare D, Piet GJ, Ulrich C, Deporte N, Egekvist J, Degel H. VMStools:

Open-source software for the processing, analysis and visualisation of fisheries logbook and

VMS data. Fish Res. 2012;115-116:31–43.

Russo T, D'Andrea L, Parisi A, Cataudella S. VMSbase: An R-Package for VMS and Logbook Data Management and Analysis in Fisheries Ecology. PLoS ONE 2014;9(6): e100195. doi:10.1371/journal.pone.0100195
