# Supplementary material for: Using Vessel Monitoring System Data to Identify and Characterize Trips Made by Fishing Vessels in the United States North Pacific
Source: PLoS One. 2016 Oct 27;11(10):e0165173. doi: 10.1371/journal.pone.0165173 (PMC5082895; doi:10.1371/journal.pone.0165173)
Supplement: S2 Text — A series of conditional statements including vessel speeds, distances from port, and time between VMS transmissions were required for determining whether vessels were in-port. Port-specific conditions are detailed here. (DOCX) [file pone.0165173.s002.docx]

**S2_Appendix**

**Approach for determination of *in-port* status for VMS records.**

Port-specific distance, speed and time thresholds were determined based on manual inspection of trips around each port. The initial steps of the trip algorithm assigned any VMS record as *in-port* if it was within 10 nmi of a port. Conditions were then used to identify which of those records should not be treated as *in-port*.

**Table S2.1**. **Port names and abbreviations used in pseudo-code for describing the *in-port* designation process for each VMS record.**

| **Port** | **Code** | **Port** | **Code** | **Port** | **Code** |
| --- | --- | --- | --- | --- | --- |
| Adak | ADA | Udagak Bay IFP | IFP | Port Lions (IFP) | PTL |
| Adak2 (IFP) | ADA2 | Juneau^†^ | JNU | Port Protection^†^ | PTP |
| Akutan | AKU | Kake^†^ | KAK | Savoonga | SAV |
| Alitak Bay | ALI | Kasilof | KAS | Seattle^‡^ | SEA |
| Angoon^†^ | ANG | King Cove | KCO | Seldovia | SEL |
| Astoria^‡^ | AST | Kenai | KEN | Seward | SEW |
| Atka | ATK | Klawock^†^ | KLA | Sitka^†^ | SIT |
| ColdBay | CDB | King Salmon | KNG | South Naknek | SNN |
| Chignik | CHG | Kodiak | KOD | Soldotna | SOL |
| Cordova^#^ | COR | Kodiak2 | KOD2 | Sanak Island (IFP) | SNK |
| Clarks Point | CPT | Ketchikan | KTN | Sand Point | SPT |
| Craig^†^ | CRG | Larsen Bay | LRB | Saint Paul | STP |
| Dillingham | DIL | Lazy Bay | LZB | Tacoma^‡^ | TAC |
| Dutch Harbor | DUT | Metlakatla^†^ | MET | Tenakee Springs^†^ | TEN |
| Egegik | EGE | Port Moller | MOL | Togiak | TOG |
| Ekuk | EKU | Naknek | NAK | Togiak2 | TOG2 |
| Elfin Cove^†^ | ELF | Ninilchik | NIN | Unalakleet | UNA |
| Emmonak | EMM | Nome | NOM | Valdez^#^ | VAL |
| False Pass | FSP | Newport^‡^ | NPT | Whittier^#^ | WHT |
| Port Graham | GRM | Nunivak Island | NUN | Wrangell^†^ | WRN |
| Gustavus^†^ | GUS | Old Harbor | OLD | Excursion Inlet^†^ | XIP |
| Hoonah^†^ | HNH | Port Alexander^†^ | PAL | Yakutat | YAK |
| Haines^†^ | HNS | Petersburg^†^ | PBG | Yantarni Bay (IFP) | YAN |
| Homer | HOM | Pelican^†^ | PEL |  |  |
| Hydaburg^†^ | HYD | Port Bailey | PTB |  |  |
|  |  |  |  |  |  |

(IFP) designates the locations of inshore fish processors, which are mobile vessels but returned to the same coordinates often enough to be treated as ports.

† Ports located in southeast Alaska

‡ Ports located in Washington or Oregon (i.e., outside of Alaska)

# Ports located in Prince William Sound

“Gaps” = refers to the time difference between the current and previous VMS record and the current and subsequent record.

“Dist” refers to distance from port in nautical miles.

“Speed” refers to the average of the forward- and backward-calculated vessel speeds.

All records were designated as *in-port* if:

- Speed < 0.1 knots & Dist < 60 nmi of NPT
- Speed < 0.1 knots & Dist < 50 nmi & Port is within southeast Alaska, Washington, Oregon, or PWS

All records were designated *at-sea* if

- PORT = (IFP, LRB, LZB, PTB, OLD, FSP, KCO, ALI, CDB, EMM, GRM, HOM, KAS, KEN, MOL, NIN, NUN, SAV, SEL, UNA) & (Dist > 2 nmi)
- PORT = CHG & (Dist > 2 nmi) & (Speed > 2 kts)
- PORT = (KCO, IFP, SPT, ATK) & (Speed ≥ 1 kts) & (Gaps ≤ 60 min)
- PORT = (KCO, ATK) & (Speed ≥ 9 kts) & (Gaps ≤ 200 min)
- PORT = FSP & (Speed ≥ 0.5 kts) & (Gaps ≤ 200 min)
- PORT = PTL & (Speed ≥ 0.5 kts) & (Dist > 2 nmi)
- PORT = SDB & ((Speed ≥ 0.5 kts & Dist > 1) or (Speed ≥ 3 kts))
- PORT = STP & ((Dist >2 nmi)) or ((Dist <2 nmi) & (Gaps < 120 min) & (Speed > 0.2 kts)))
- PORT = KOD & ((Dist > 5 nmi) or ((Dist > 1 nmi) & (Gaps < 60 min) & (Speed > 2 kts)))
- PORT = TOG & (Speed > 2 kts)
- PORT = TOG2 & (Speed > 2 kts) & (Dist > 10 nmi)
- PORT = SNK & ((Speed > 0.5 & Dist > 1.5 nmi) or (Speed > 5))
- PORT = AKU & (((Speed ≥ 9 kts) & (Gaps < 35 min)) or ((Speed ≥ 5 kts) & (Gaps < 60 min)))
- PORT = ATK & (Gaps < 30 min) & (Speed > 0.5 kts)
- PORT = (ADA, ADA2, YAN) & (Speed ≥ 0.1 kts) & (Dist > 2 nmi)
- PORT = SPT & (Dist > 7 nmi) & (Speed > 0.1 kts))
- PORT = (FSP, DUT, KOD, AKU) & the total duration of an *in-port* period is < 120 min
- Gaps > 3000 min

Accounting for gaps in VMS transmission > 120 min

Several contingencies were necessary to account for cases during which large gaps in VMS transmissions occurred between the last *at-sea* record and the first *in-port* record. If more than 120 min^^[[1]](#footnote-1)^^ passed between these two VMS records, then the two were decoupled (i.e., the first *in-port* record was not considered to be part of the trip that was ending). The same methodology was applied to the last *in-port* record of a cluster and the start of the subsequent trip.

If only one *in-port* record existed in a cluster and the time difference between that record and one of the *at-sea* clusters on either side was > 120 min, then the *in-port* record was assigned to the trip < 120 min away. If both trips were > 120 min then neither trip was coupled to the *in-port* record. If both trips were < 120 min from the *in-port* record, the prior of the two trips was (arbitrarily) coupled to the *in-port* record.

Using VMS from observed trips, we examined the distribution of long gaps in VMS transmissions that occurred while a vessel was *at-sea*. Long gaps were relatively rare during trips (as opposed to while a vessel was in / near port) and we qualitatively determined 3,000 min (i.e., 50 hours) to be the maximum allowable gap threshold during a trip. Any gap between VMS records > 3,000 min automatically triggered the start of a new trip.

1. The 120 min threshold was chosen after inspection of gaps during many trips and based on the minimum time in port for most trips. [↑](#footnote-ref-1)
