# Supplementary material for: Using Vessel Monitoring System Data to Identify and Characterize Trips Made by Fishing Vessels in the United States North Pacific
Source: PLoS One. 2016 Oct 27;11(10):e0165173. doi: 10.1371/journal.pone.0165173 (PMC5082895; doi:10.1371/journal.pone.0165173)
Supplement: S3 Text — Fish tickets and VMS records do not contain timestamps on the same scale and fish tickets dates are manually recorded and can involve complicated multi-day or multi-port deliveries. We detail the matching of these two datasets here. (DOCX) [file pone.0165173.s003.docx]

**S3_Appendix**

**Fish ticket matching**

Fish ticket matching proceeded via a series of conditional statements dependent upon a VMS record falling within a reported statistical area during the fish ticket-reported dates of the trip. Some fish tickets reported catch that was landed during a single delivery (“Complete delivery trips”) while others (~ 1% of fish tickets) reported partial deliveries (“Partial delivery trips”) during which catch was landed at multiple processors or over multiple days. Slightly different matching protocols were used for the complete and partial delivery trips.

Terms:

*VMS start*: start date of a trip based on VMS

*VMS end*: end date of a trip based on VMS

*FT start*: fish ticket-reported date during which fishing started (reported individually for each statistical area in which a vessel fished during a trip)

*FT end*: fish ticket-reported date during which catch was landed

VMS trip numbers were linked to fish tickets when a VMS record fell within one of the statistical areas reported on the fish ticket between (inclusive) the *FT start* and *FT end*.

Complete delivery trips

- Many duplicate matches occurred when one trip ended on the same day that fishing from the subsequent trip began.
  - If among the duplicate matches, the *VMS start* and *VMS end* perfectly matched the *FT start* and *FT end* dates of only one of the fish ticket trips, assign the match to that trip.
  - If among the remaining duplicate matches, the *VMS start* and *FT start* match and the *VMS end* ≤ *FT end*, assign the match to that trip
  - If among the remaining duplicate matches, the *VMS start* < *FT start* and the *VMS end* ≤ *FT end*, then assign the match to that trip.
- Erroneous matches may have occurred if ((*FT start* < *VMS start*) and (*FT end* equals *VMS start*) and *FT end* < *VMS end*) or ((*FT start* > *VMS start*) and (*FT start* equals *VMS end*) and *FT end* > *VMS end*). In such cases remove the match (it will be matched elsewhere).

Partial delivery trips

- Create a unique identifier (ID) for each *FT start* and *FT end* combination.
- With the fish tickets sorted chronologically, create a set of lagged date fields such that each row also contains the subsequent *FT start* and *FT end*.
- Match subsequent fish tickets with the same *FT start* and *FT end* to the same VMS trip.
- Of the remaining rows for which VMS/FT entries have some overlap, assign each of the overlapping groups of rows a cluster ID (i.e., at least two rows per ID value).
- For each cluster ID value, tally the number of unique VMS trip IDs, the number of FT start dates and the number of different FT landing dates.
- If a cluster ID only has one FT landing date then the entries for that date are simply one trip with ≥ 1 start date. These are analogous to non-partial delivery trips and are extracted and stored.
- If a cluster has multiple start and multiple landing dates, then extract those for which the landing date of the first FT is equal to the start date of the subsequent FT (and for which the start and landing dates are not the same for the first trip). In this case, a trip ends on the same day that the next trip starts but the VMS trip should be matched to the first of the two fish tickets. Extract and store the former of the VMS/FT matches here.
- If for a cluster ID, there is a single FT start date and only one VMS trip value and the first FT landing date is < the subsequent FT landing date, then the VMS trip ended during the first FT landing date (in such cases, the vessel probably delivered multiple batches but over two days, without leaving port. Thus the second of the deliveries is not associated with a VMS trip). The former VMS trip / FT match is retained.
- For the matched trips, we now need to rejoin these values with the duplicated ID3 values that had been removed before - this will return all of the stat areas for a given trip.
