# Supplementary material for: Using Vessel Monitoring System Data to Identify and Characterize Trips Made by Fishing Vessels in the United States North Pacific
Source: PLoS One. 2016 Oct 27;11(10):e0165173. doi: 10.1371/journal.pone.0165173 (PMC5082895; doi:10.1371/journal.pone.0165173)
Supplement: S4 Text — Time and distances traveled while vessels are in-port can affect the comparison between observed and VMS-based trip durations. Here we describe the process of identifying offset values to account for such behaviors. (DOCX) [file pone.0165173.s004.docx]

**S4_Appendix**

**Calculation of distances traveled and durations spent in transit while a vessel was *in-port*.**

We standardized *in-port* durations and distances traveled for Dutch Harbor and Akutan by examining trip starts where the VMS data had gaps ≤ 30 min from the time the vessel left the dock and the time the vessel reached the 10 nmi threshold (and vice versa for the end of trips). We considered a vessel to have just left the dock if the speed between the current and previous VMS record was < 0.25 knots and the speed between the current and subsequent record was > 0.25 knots. Records at the beginning of a trip that met these criteria (Dutch Harbor N=35 trips; Akutan, N=1,081 trips) were then compared with the first VMS record at-sea. If the first at-sea record was exactly 10 nm from port, the duration and distances traveled between that point and the first record away from the dock were calculated. In most cases, the first *at-sea* record occurred > 10 nmi from port. Using the speed calculated between the two VMS records straddling the 10 nmi boundary, the point at which the boundary was crossed was linearly interpolated. The time and distance traveled between the dock and the interpolated point were subsequently determined. The reverse process was performed at the end of trips, identifying instead the last record *at-sea* and calculating the distance and duration traveled until the last record when the previous speed was > 0.25 knots and the subsequent speed was < 0.25 knots (Dutch Harbor N=13 trips; Akutan N=1,200 trips).

Trips in Dutch Harbor spent an average of 80 min and traveled 10 nm while *in-port* with the same amount of time (t-test, P > 0.1) and distances (t-test, P > 0.1) spent at both the beginning and the end of the trip. Akutan trips differed between the start and ends of trips for both duration (t-test, P < 0.01) and distance traveled (t-test, P < 0.01). Akutan trips began with an average of 101 min and 13 nm of transit and ended with 92 min and 12 nmi of transit.

In order to apply the constant *in-port* duration and distances consistently across all Dutch Harbor and Akutan trips, we also had to standardize the points to which the constant start and end values were appended. If the first *at-sea* record occurred at exactly 10 nmi from port, the constant could simply be added to the time and distance of that VMS record. More often however, the last *in-port* and first *at-sea* records straddled the 10 nmi threshold (e.g., records were 8 nmi and then 12 nmi from port). In such cases, we linearly interpolated the point at which the vessel would have crossed the 10 nmi threshold based on the calculated speed between the two records on either side of the threshold. The *in-port* constants were then added to that interpolated point.

In some cases (~12% of trips), no *in-port* records existed for a trip and the first (or last) VMS record was outside the 10 nmi threshold. In such instances, the first record could occur anywhere between 10 nmi and several hundred nautical miles from port. Vessel trajectories were extrapolated between the first (or last) VMS record back to port using a transit speed of 8.5 knots^^[[1]](#footnote-1)^^.

1. The mean speed of observed vessels transiting between 10 and 50 nmi from port was 8.1 knots. The median speed over this same period was 8.9 knots. We used the average of these two speed values. [↑](#footnote-ref-1)
