# Supplementary material for: Using Vessel Monitoring System Data to Identify and Characterize Trips Made by Fishing Vessels in the United States North Pacific
Source: PLoS One. 2016 Oct 27;11(10):e0165173. doi: 10.1371/journal.pone.0165173 (PMC5082895; doi:10.1371/journal.pone.0165173)
Supplement: S5 Text — We explore regressions to estimate and correct for bias in the estimation of trip duration as compared to observer data. (DOCX) [file pone.0165173.s005.docx]

**S5_Appendix**

**Regression to correct estimated trip duration**

We examined a series of linear, generalized linear and generalized additive modeling approaches to best fit observed trip duration with covariates including vessel, VMS-based trip duration, season (the pollock season is divided into a winter / spring season and a summer/ fall season), year, the distances from port of the first and last VMS records of a trip, and the size of gaps in VMS transmissions. Model selection was based on predictive ability, so models were compared by fitting a randomly selected subset of 75% of trips and comparing prediction accuracies from the remaining 25%. This model comparison was based on their ability to predict with the lowest percent error ([observed – predicted]/observed) for several different metrics:

1. *Aggregate* (aggregate error), the error of the sums of all trip durations where positive errors indicate under-estimation.

$$\frac{100*\sum_{i=1}^{i} {Observed duration}_{i}-\sum_{i=1}^{i} {Predicted duration}_{i}}{\sum_{i=1}^{i} {Observed duration}_{i}}$$

1. *Trip* (trip-level error), where the mean / median was calculated for the percent errors

of all trips. This metric assesses whether there is a bias in the predicted duration (e.g., a positive value would indicate that, on average, the model under-predicted duration):

$$100*mean\left( \frac{{Observed duration}_{i}-{Predicted duration}_{i}}{{Observed duration}_{i}} \right)$$

1. *Abs* (mean/median absolute trip-level error), similar to the trip-level error but assesses the magnitude of the errors instead of the bias:

$$100*mean\left| \frac{{Observed duration}_{i}-{Predicted duration}_{i}}{{Observed duration}_{i}} \right|$$

A suite of GAM and linear models were tested with covariates including: estimated trip duration, pollock fishing season, vessel size, the distances from port of the first (*startDIST)* and last (*endDIST*) VMS records per trip, number of VMS records per trip, the mean VMS transmission interval, and year. GAMs had consistently lower prediction errors for all three error metrics than did linear models. A back-fitting process was used for model selection but instead of comparing AIC from successive removals of predictors, we compared the change in the prediction accuracy. If a predictor was removed without decreasing the prediction accuracy, the predictor was dropped from the model. This process was repeated with both default smoothing of the predicted duration term and with the estimated degrees of freedom constrained to 4 (to determine if over-fitting was a factor in the prediction accuracy).

Exploratory plots revealed a clear break in trip durations (Fig 1b) greater than or less than 700 min. Because vessel behaviors may vary between short and long trips, we explored model selection for models that included all data, data for trips < 700 min, and data for trips ≥ 700 min. While fit with different coefficients, the latter two models were combined into our so-called piecewise model.

Model selection using three different error metrics was straightforward for most models but a final set of models had either similar accuracy or their performances varied across metrics, making a decision more difficult. Thus we present three final models (Table S5.1), each fit to the full dataset and to the two piecewise datasets. For comparison, we also provide the errors from the raw data (i.e., observed vs. VMS-estimated trip duration).

A final set of best models was iteratively fit to 100 randomly sampled training datasets consisting of 60% of the total data (additional models with different training-test data splits were also examined – results not shown) and tested on the remainder of data for each dataset and iteration. Training and test dataset sizes were varied to examine how results may fluctuate for fisheries with different levels of observer coverage.

We present results for a GAM that included only the estimated duration as a predictor, and a GAM that included several predictors:

ln(Duration_observed_) = α + s_1_(ln(Duration_estimated­_)) + s_2_(startDIST) + s_3_(endDIST) + ε,

where *s(·)* are smooth functions of the VMS-estimated trip duration (Duration_estimated­_), and the distances from port of the first (*startDIST)* and last (*endDIST*) VMS records per trip. Smooth functions were estimated via thin plate regression splinesTable S5.1 includes errors from the above GAM with automatic smoothing selection (GAM1), a GAM with restricted smoothing on estimated duration (edf=4; GAM2), and a GAM with only ln(Duration_estimated_) as a covariate (GAM3).

Regression efforts to standardize estimated trip duration yielded mixed results, depending on whether we were more interested in aggregate trip durations or trip-level durations. For the former, GAMs reduced the aggregate percent error by more than half in some cases (e.g., GAM1 and GAM2, or GAM3 with the single regression). However, in most cases the trip-level errors were lower without using the regression. For those models that were successfully fit, prediction accuracies were similar across the range of test dataset sizes.

**Table S5.1.** **Percent errors (±1 SD) from model adjusted and unadjusted trip durations.** Models were run on both a single set of duration data and via a piecewise regression that modeled trips ≥ or < 700 minutes separately and combined their predictions. *Aggregate* is the percent error between the sum of observed trip durations and the sum of predicted trip durations (positive values represent under-prediction). *Trip* is either the mean or median of all the percent differences between the VMS-estimated and the observed duration for each trip (positive values represent under-prediction). *Abs* takes the absolute value of the percent differences in *Trip* prior to summarizing with the mean or median (all values are positive).

|  |  |  | **Single regression** | **Piecewise regression** | **Single regression** | **Piecewise regression** | **Single regression** | **Piecewise regression** |
| --- | --- | --- | --- | --- | --- | --- | --- | --- |
|  |  | No model | GAM1 | GAM1 | GAM2 | GAM2 | GAM3 | GAM3 |
| **Training Data - 60%** | Aggregate | 1.40(0.1) | 0.58(0.2) | 0.48(0.2) | 0.59(0.2) | 0.5(0.2) | 0.57(0.2) | 1.44(0.2) |
|  | TripMean | 0.34(0.3) | -1.27(0.4) | -0.77(0.2) | -1.24(0.4) | -0.75(0.2) | -1.31(0.4) | -1.19(0.3) |
|  | TripMedian | -0.03(0.0) | -0.88(0.1) | -0.65(0.1) | -0.92(0.1) | -0.64(0.1) | -0.93(0.1) | -1.04(0.2) |
|  | AbsMean | 5.78(0.2) | 6.78(0.2) | 5.71(0.1) | 6.79(0.2) | 5.55(0.1) | 6.35(0.3) | 7.93(0.2) |
|  | AbsMedian | 1.27(0.0) | 2.63(0.1) | 2.17(0.1) | 2.72(0.1) | 1.94(0.1) | 1.73(0.1) | 4.33(0.2) |
