# Supplementary material for: Using Vessel Monitoring System Data to Identify and Characterize Trips Made by Fishing Vessels in the United States North Pacific
Source: PLoS One. 2016 Oct 27;11(10):e0165173. doi: 10.1371/journal.pone.0165173 (PMC5082895; doi:10.1371/journal.pone.0165173)
Supplement: S6 Text — We describe the conditions used to identify trips as fishing or non-fishing and for those trips identified as fishing, we further assign them as fishing for pollock in the Bering Sea or having other targeting behavior. (DOCX) [file pone.0165173.s006.docx]

**S6_Appendix**

**Characterizing fishing and non-fishing trips**

Fish tickets only exist for fishing trips and thus, all trips that were matched with a fish ticket were classified as fishing trips. Fishing trips were designated as non-AFA trips if fish ticket codes identified management other than “AFA”, gear other than pelagic trawl (the only gear allowed by the AFA), fishing outside of the Bering Sea (NMFS areas 500 – 530; https://alaskafisheries.noaa.gov/sites/default/files/reporting-areas.pdf), or delivery to a non-AFA processor. Fishing trips were also designated as non-AFA if their entirety occurred outside of the Bering Sea or outside of the AFA pollock fishing seasons (https://alaskafisheries.noaa.gov/sustainablefisheries/plckseas.pdf), or if the trip landed at St. Paul Island (which has no AFA processor) without a fish ticket from an AFA floating processor. Trips with observer data from the west coast fishery for Pacific hake (*Merluccius productus*) were also listed as non-AFA.

Much of the geographic range of the AFA CV fleet can be generally designated as consisting of non-fishing trips. Vessel activities each summer may involve tendering for salmon fisheries in Bristol Bay^^[[1]](#footnote-1)^^, southeast Alaska or Prince William Sound (PWS). Additionally, many vessels’ homeports and maintenance yards are in Washington State or Oregon and trips may include long transits between Alaska and the Pacific Northwest. We designated any trip (without a fish ticket) that originated or terminated in one of these regions (Bristol Bay, southeast Alaska, PWS, Pacific Northwest) (Table S6.1), as well as trips that occurred while a vessel was under charter^^[[2]](#footnote-2)^^, as non-fishing trips. Some vessels may participate in a state-managed PWS groundfish fishery between January and March, so PWS trips during this time which landed in either Kodiak or Seward were designated as non-AFA fishing trips; all other PWS trips were designated as non-fishing.

**Table S6.1** **Ports within each region.** Port names are listed by the North Pacific region into which they are grouped for use with the algorithm. All trips that start or end in BB, PNW, PWS, or SEAK are designated as non-fishing trips. All KOD ports may represent non-fishing trips or non-AFA fishing trips.

| **Bristol Bay (BB)** | **Kodiak (KOD)** | **Pacific Northwest (PNW)** | **Prince William Sound (PWS)** | **Southeast Alaska (SEAK)** |
| --- | --- | --- | --- | --- |
| Clark’s Point | Alitak Bay | Anacortes, WA | Cordova | Craig |
| Dillingham | Larson Bay | Astoria, OR | Valdez | Elfin Cove |
| Egegik | Lazy Bay | Bellingham, WA | Whittier | Gustavus |
| Ekuk | Nelson Bay | Blaine, WA |  | Hoonah |
| South Naknek | Old Harbor | Newport, OR |  | Juneau |
| Ugashik | Port Bailey | Seattle, WA |  | Ketchikan |
|  |  | Tacoma, WA |  | Pelican |
|  |  | Vancouver, BC |  | Petersburg |
|  |  |  |  | Pt. Alexander |
|  |  |  |  | Sitka |
|  |  |  |  | Tenakee |
|  |  |  |  | Wrangell |
|  |  |  |  | Excursion Inlet |
|  |  |  |  | Yakutat |

Within the Bering Sea, there were two vessel transit “corridors” that typically included short, non-fishing trips between Dutch Harbor and Akutan (~ 45 nmi travel distance) or between Dutch Harbor and a floating processor in nearby Beaver Inlet (~ 33 nmi travel distance). Any trip for which all VMS records were contained within one of these corridors was designated as non-fishing.

Trawl gear used by North Pacific groundfish fisheries was typically fished at speeds between ~ 1 - 5 knots so trips without at least one VMS records < 5 knots while the vessel was *at-sea*^^[[3]](#footnote-3)^^ were unlikely to be fishing^^[[4]](#footnote-4)^^. To be conservative, any trip (without a fish ticket) whose minimum *at-sea* speed was > 6 knots were designated as a non-fishing trip.

An additional filter identified all state management areas in which fishing was observed. We examined the minimum vessel speed within any of these management areas for the duration of a trip. If the minimum speed in one of these areas never fell between 0.5 - 5 knots, it was deemed a non-fishing trip. If the speed only fell within this threshold for management areas that were outside of the Bering Sea region, that trip was identified as a non-AFA fishing trip.

Non-AFA fishing grounds were defined as those state statistical areas in which at least 50 VMS records occurred during observed fishing. We calculated the mean vessel speed across all of these areas. If a trip included at least one of these areas and the mean vessel speed within that area was 0.5 - 5 knots, the trip was designated as a fishing trip. Many mean speeds within fishing areas were > 5 knots because a vessel may transit through one fishing area while traveling to another. However, it is notable how many mean speeds are < 5 knots for fishing trips and equally notable how few mean speeds for non-fishing trips were < 5 knots (Figure S6.1). Several non-fishing trips did have mean speeds < 0.5 knots and manual inspection of these trips usually indicated that a vessel was anchored overnight. Only 2.6% of the 980 non-fishing trips fell within this speed range; 99.6% of the known fishing and non-fishing trips in these areas (N=5,573) were correctly characterized based on this speed filter. All of the areas examined here were outside of the AFA fishery boundaries so all of these trips designated as fishing were also determined to be non-AFA fishing trips.

Finally, all fishing trips should have a fish ticket. So while any remaining fishing trips were unmatched to a fish ticket (likely because of incorrect date-reporting on the fish ticket or VMS gaps), an analysis of the fish ticket data alone should reveal whether or not a vessel’s trips were AFA or non-AFA. For a given month-year combination, if a vessel only had fish tickets from AFA deliveries, then any fishing trip during that vessel’s month-year combination was considered to be an AFA trip (or vice versa for non-AFA deliveries).

The GAM used to predict fishing versus non-fishing relied on regional groupings for both start and end port (Table S6.2). The groupings from Table S6.1 were further consolidated such that only 4 regions were included in the model for start and end port.

**Table S6.2**. **Port names and abbreviations used in pseudo-code for describing the *in-port* designation process for each VMS record.**

| **Gulf of Alaska** | **Bering Sea** | **Aleutian Islands** | **Other^*^** |
| --- | --- | --- | --- |
| Cold Bay | Akutan | Adak | Southeast Alaska |
| Chignik | Dutch Harbor | Atka | Bristol Bay |
| False Pass | Floating Processor | Adak2 (floating processor) | Prince William Sound |
| Homer |  |  | Pacific Northwest |
| King Cove |  |  |  |
| Ninilchik |  |  |  |
| Seward |  |  |  |
| Sand Point |  |  |  |
| Yantarni Bay |  |  |  |

See Table S6.1 for port groupings

Model selection was primarily based on predictive ability but model output is provided in Table S6.3, nonetheless.

**Table S6.3** Model output for GAM used to predict whether fishing occurred on a trip.

| Parametric coefficients | Estimate | Std. Error | Z value | Pr(>\|z\|) |
| --- | --- | --- | --- | --- |
| (Intercept) | 3.76 | 0.27 | 13.94 | <<0.01 |
| SEASON-B | -0.19 | 0.17 | -1.14 | 0.256 |
| SEASON-N | -0.77 | 0.29 | -2.66 | 0.008 |
| START.Bering | 0.03 | 0.25 | 0.11 | 0.912 |
| START.Aleutian | -2.49 | 0.56 | -4.46 | <<0.01 |
| START.Other | -0.54 | 0.90 | -0.60 | 0.55 |
| END.Bering | 0.69 | 0.25 | 2.81 | 0.005 |
| END.Aleutian | 2.48 | 0.53 | 4.69 | <<0.01 |
| END.Other | 3.87 | 0.78 | 4.98 | <<0.01 |
|  |  |  |  |  |
| Smooth terms |  |  |  |  |
|  | edf | Ref.df | Chi.sq | p-value |
| s(lDuration,avspstat) | 20.9 | 24.9 | 696.4 | <<0.01 |
| s(sddif) | 4.5 | 5.4 | 77.0 | <<0.01 |
| s(sdsp) | 6.2 | 7.3 | 200.2 | <<0.01 |
|  |  |  |  |  |
| R^2 (adj) | 0.9 |  |  |  |
| Deviance explained | 91.10% |  |  |  |


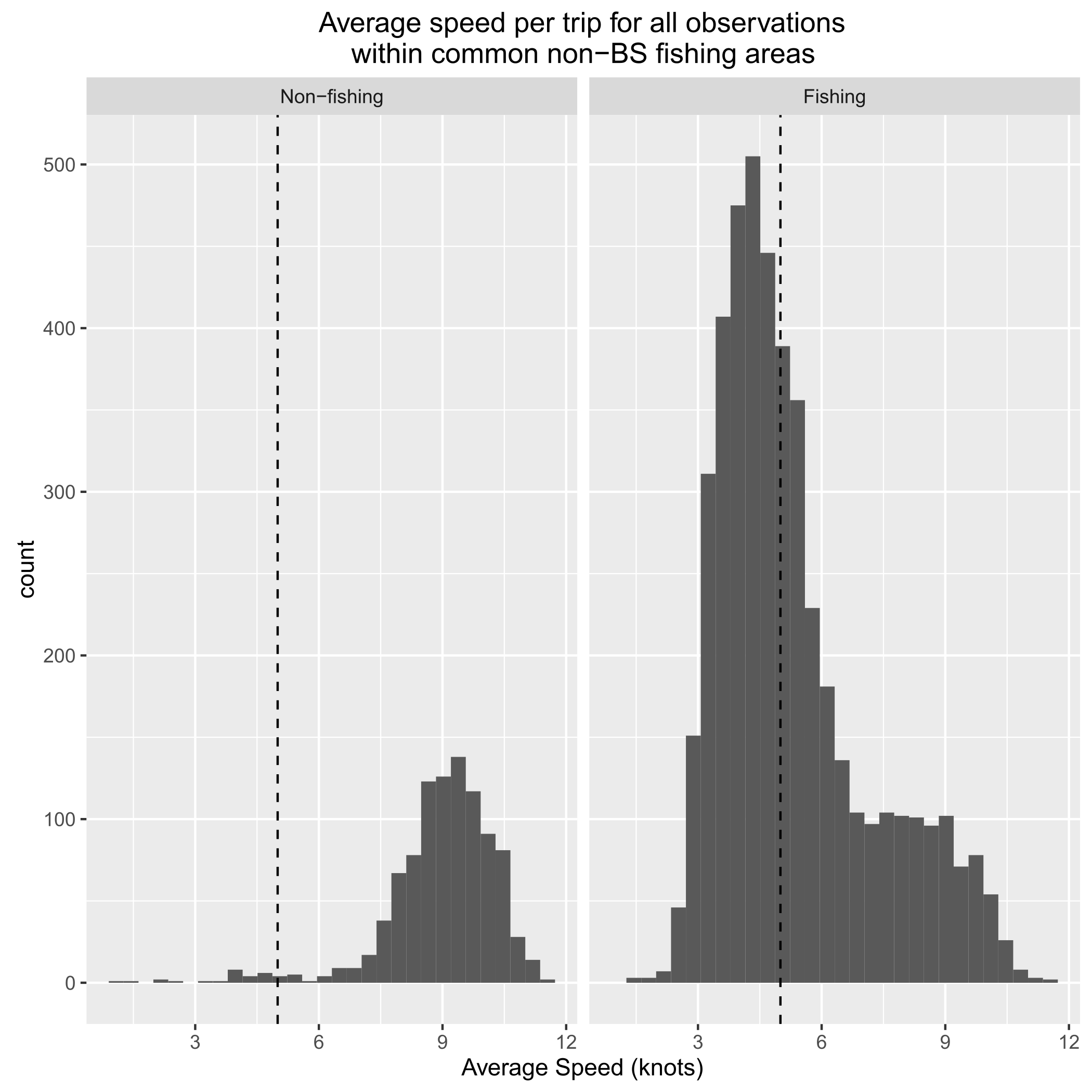


Figure S6_1. **Average speed of VMS records that lie within commonly fished non-Bering Sea fishing grounds.** Illustration that the majority of VMS records from non-fishing trips have average speeds > 5 knots (vertical dashed line) while in common fishing areas outside of the Bering Sea. Fishing trips, however are much more likely to have slow speeds (< 5 knots).

1. Ports of Ekuk, Egegik, Dillingham, Naknek, Togiak; pollock fishing is prohibited in this area [↑](#footnote-ref-1)
2. Vessels may be chartered for research surveys by NMFS. [↑](#footnote-ref-2)
3. >10 nmi from the nearest port in this case, to add a conservative buffer. [↑](#footnote-ref-3)
4. Approximately 0.2% of observed fishing trips had a minimum *at-sea* speed > 6 knots; these trips were characterized by large gaps in VMS transmissions that may have omitted slower speeds. [↑](#footnote-ref-4)
