# Supplementary material for: Using Vessel Monitoring System Data to Identify and Characterize Trips Made by Fishing Vessels in the United States North Pacific
Source: PLoS One. 2016 Oct 27;11(10):e0165173. doi: 10.1371/journal.pone.0165173 (PMC5082895; doi:10.1371/journal.pone.0165173)
Supplement: S7 Text — We describe the process of spatial matching that identifies non-fishing transit corridors between the ports of Dutch Harbor and Akutan and between Dutch Harbor and a floating processor. (DOCX) [file pone.0165173.s007.docx]

**S7_Appendix**

**Non-fishing corridor between Dutch Harbor and Akutan or Beaver Inlet**

The majority of personnel and supplies for the port of Akutan are transported from Dutch Harbor via fishing vessels and thus many trips occur between these two ports (~ 43 nmi) (Fig S7.1). Often, vessels will leave Dutch Harbor and stop in Akutan before then heading to the fishing grounds. Similarly, vessels may deliver their catch in Akutan before then returning to Dutch Harbor prior to their next fishing trip. Arguably, many of the transits between Dutch Harbor and Akutan are thus part of a fishing trip but for consistency, we define these trips as non-fishing trips^^[[1]](#footnote-1)^^.

To define the corridor, we plotted VMS tracks from observed non-fishing transits between Dutch Harbor and Akutan and manually^^[[2]](#footnote-2)^^ created a polygon shapefile that encompassed the VMS records from these trips (*rgdal* Version 0.9-1 and *sp* Version 1.0-16 packages in R Statistical Software Version 3.1.1). This polygon subsequently served as an *ad hoc* transit corridor; any trip for which all VMS records fell within this polygon were determined to be transiting between Dutch Harbor and Akutan and were thus designated as *non-fishing*.

A similar transit situation exists between a floating processor located in Beaver Inlet (Fig S7.1), an approximately 33 nm transit from Dutch Harbor. Most transits occur after a vessel has delivered their catch to the floating processor and then returns to Dutch Harbor. Because the Observer Program considers floating processors to be “ports,” observers end a trip when a vessel ties up at a floating processor, and thus so does our approach. Subsequently, the transit from the floating processor back to Dutch Harbor is a standalone, non-fishing trip. The same approach was followed for creating a polygon shapefile that encompasses entire trips transiting between Dutch Harbor and this stationary floating processor.


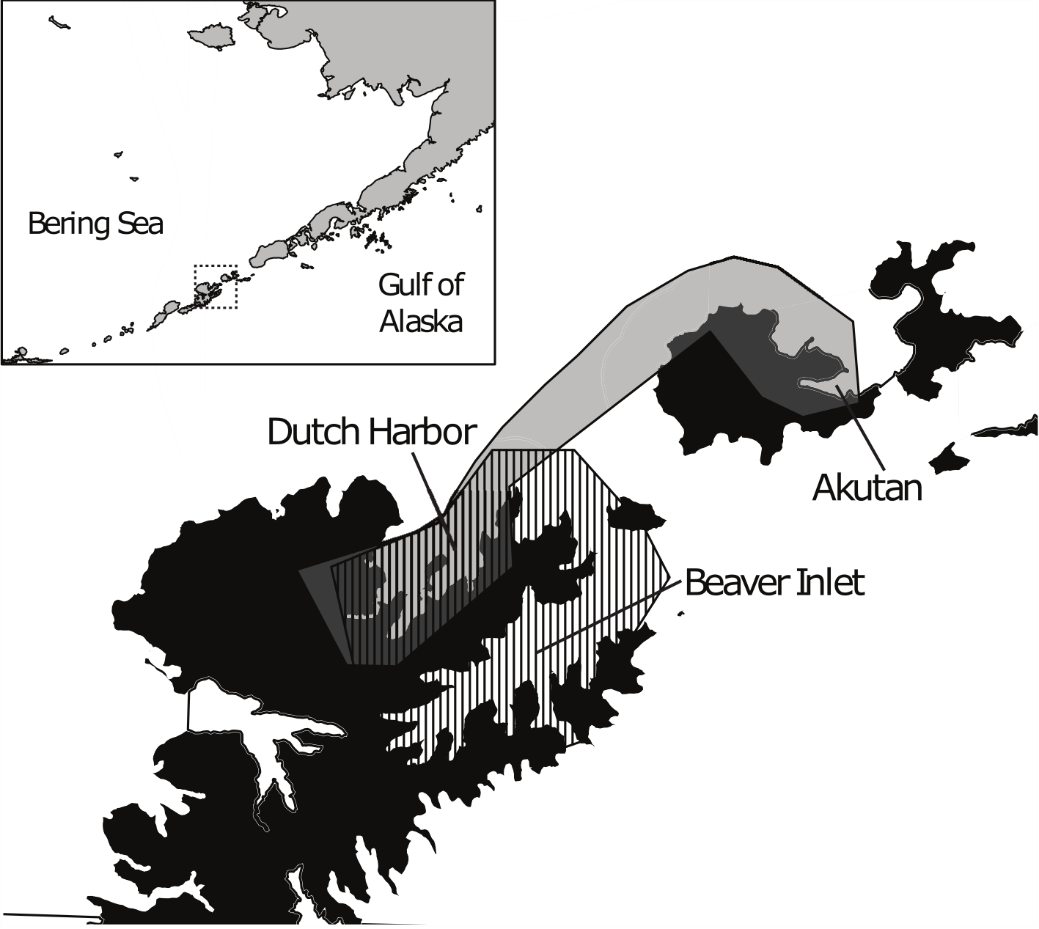


Figure S7_1. **Transit corridors near Dutch Harbor**. Illustration of the corridors of non-fishing transit trips between Dutch Harbor and Akutan (shaded grey) and Dutch Harbor and Beaver Inlet (hatched vertical lines).

1. Vessels may make no fishing trip between Dutch Harbor / Akutan transits, they may make a single trip to the fishing grounds, they may make repeated trips to the fishing grounds from Akutan before returning to Dutch Harbor, or they may visit another port prior to revisiting Dutch Harbor or Akutan. [↑](#footnote-ref-1)
2. Note that a convex hull function was not used here in order to provide a buffer for future points that may lie slightly “on-land” as was discussed in the Methods section on matching VMS records with the nearest NMFS management area. [↑](#footnote-ref-2)
