# Supplementary material for: Using Vessel Monitoring System Data to Identify and Characterize Trips Made by Fishing Vessels in the United States North Pacific
Source: PLoS One. 2016 Oct 27;11(10):e0165173. doi: 10.1371/journal.pone.0165173 (PMC5082895; doi:10.1371/journal.pone.0165173)
Supplement: S8 Text — We present the distribution of trip types and ports as a complement to Table 2. (DOCX) [file pone.0165173.s008.docx]

**S8_Appendix**

**Distribution of fishing and non-fishing trip types and ports**

As a complement to Table 3 (main body of manuscript), the distribution of trip types and ports are presented. There were 19,302 fishing trips (12,280 AFA and 7,022 non-AFA) and 10,494 non-fishing trips. Among the non-fishing trips, 296 were surveys, 1,515 remained within in the corridor between Dutch Harbor and Beaver Inlet, 2,106 remained within the corridor between Dutch Harbor and Akutan, and 5,422 occurred within Bristol Bay, Prince William Sound, Southeast Alaska, or the Pacific Northwest. The remainder were scattered throughout the BSAI and GOA.

Among the non-AFA fishing trips, 10.7% landed their catches in the Aleutian Islands, 32.2% landed in the Bering Sea (Dutch Harbor, Akutan and a few trips at St. Paul Island), 49.6% landed their catches in Kodiak, with the remaining 7.5% of trips landing elsewhere in the GOA. AFA fishing trips were primarily split between Akutan (39.2%) and Dutch Harbor (45.3%), with the remainder going to the Beaver Inlet floating processor (10.5%), King Cove (4.0%) and a few other processors and motherships.
